# *IsoSearch*: An Untargeted and Unbiased Metabolite and Lipid Isotopomer Tracing Strategy from HR-LC-MS/MS Datasets

**DOI:** 10.3390/mps3030054

**Published:** 2020-07-30

**Authors:** He Huang, Min Yuan, Phillip Seitzer, Susan Ludwigsen, John M. Asara

**Affiliations:** 1 Division of Signal Transduction, Beth Israel Deaconess Medical Center, Boston, MA 02115, USA; he_huang@fudan.edu.cn (H.H.); myuan@bidmc.harvard.edu (M.Y.); 2 Department of Medicine, Harvard Medical School, Boston, MA 02115, USA; 3 Institute of Metabolism and Integrative Biology, Fudan University, Shanghai 200438, China; 4 Proteome Software, Inc., Portland, OR 97219, USA; phillipseitzer@gmail.com (P.S.); susan.ludwigsen@proteomesoftware.com (S.L.); 5 Calico Life Sciences, San Francisco, CA 94080, USA

**Keywords:** stable isotope labeling, flux, isotopic tracer analysis, polarity switching, high resolution, liquid chromatography, mass spectrometry, ^13^C, ^15^N, glucose, glutamine, cancer, metabolism, metabolomics, lipidomics, cell culture, LC-MS/MS

## Abstract

Stable isotopic tracer analysis is a technique used to determine carbon or nitrogen atom incorporation into biological systems. A number of mass spectrometry based approaches have been developed for this purpose, including high-resolution tandem mass spectrometry (HR-LC-MS/MS), selected reaction monitoring (SRM) and parallel reaction monitoring (PRM). We have developed an approach for analyzing untargeted metabolomic and lipidomic datasets using high-resolution mass spectrometry with polarity switching and implemented our approach in the open-source R script IsoSearch and in Scaffold Elements software. Using our strategy, which requires an unlabeled reference dataset and isotope labeled datasets across various biological conditions, we traced metabolic isotopomer alterations in breast cancer cells (MCF-7) treated with the metabolic drugs 2-deoxy-glucose, 6-aminonicotinamide, compound 968, and rapamycin. Metabolites and lipids were first identified by the commercial software Scaffold Elements and LipidSearch, then IsoSearch successfully profiled the ^13^C-isotopomers extracted metabolites and lipids from ^13^C-glucose labeled MCF-7 cells. The results interpreted known models, such as glycolysis and pentose phosphate pathway inhibition, but also helped to discover new metabolic/lipid flux patterns, including a reactive oxygen species (ROS) defense mechanism induced by 6AN and triglyceride accumulation in rapamycin treated cells. The results suggest the IsoSearch/Scaffold Elements platform is effective for studying metabolic tracer analysis in diseases, drug metabolism, and metabolic engineering for both polar metabolites and non-polar lipids.

## 1. Introduction

Metabolic flux analysis (MFA) using stable isotope tracers is a technique used to investigate the intracellular metabolic rate of cells and organisms [1,2]. Using nuclear magnetic resonance (NMR) or mass spectrometry (MS), steady-state metabolite flux in biological systems has been widely studied in the past few decades [3,4,5]. Although unlabeled steady-state metabolomics profiling can provide information on metabolite level alterations, the directionality associated with metabolic fluxes is not easily obtained [6]. Stable isotope labeled nutrients, such as ^13^C-glucose and ^15^N-glutamine, are frequently used in targeted mass spectrometry (GC-MS or LC-MS) based approaches to analyze metabolite isotopomer incorporations [1,6,7]. In particular, selected reaction monitoring (SRM) is often used to detect the heavy isotope labeled metabolites identified by their unique precursor ion and fragment ion transitions based on the number of labeled carbons. The SRM based targeted flux analysis built in our lab has been used to profile central carbon metabolism in various diseases. In references [8,9,10,11,12,13] However, like most existing MFA approaches, targeted fluxomics are limited to selected metabolite reactions in defined metabolic pathways (such as central carbon metabolism, amino acid metabolism, nucleotides, etc.); thus, a global metabolic fluxomics method is needed to discover new metabolic reactions from alternative pathways including minor metabolic contributions from reaction intermediates. Many laboratories have developed various software tools to interpret the MFA results generated by NMR [14], GC-MS [15,16,17], or LC-MS, such as SUMOFLUX [18], ^13^C-FLUX [14,19], X^13^CMS [20], and Omix [21]. SUMOFLUX is a MATLAB based program that uses surrogate modeling and machine learning techniques to analyze targeted ^13^C-metabolic flux models. ^13^C-FLUX is a toolbox that takes GC-MS data or converted NMR data for targeted metabolic flux analysis [15]. The ^13^C-FLUX results can also be visualized by the Omix software. Although both SUMOFLUX and ^13^C-FLUX can process targeted metabolic flux results, they are less powerful for managing untargeted fluxomics results and discovering new metabolic flux mechanisms. X^13^CMS aims to analyze untargeted metabolic isotopic data by requiring a paired isotope labeled experiment with an unlabeled sample, and differentiates the isotopomers based on the mass differences. Using X^13^CMS, several new carbon source fluxes were discovered, such as itaconate flux in the polarized macrophages [22], acetoacetate shuttles between the hepatocyte and macrophage [23], and mammalian mitochondrial lactate flux [24]. However, it is not always possible to obtain the paired isotopic experiment samples required by X^13^CMS due to acquisition conditions. Moreover, the dependence on XCMS outputs makes X^13^CMS unlikely to incorporate results from other software such as Proteome Software’s Scaffold Elements [25] and Thermo’s LipidSearch.

**Figure 1:**
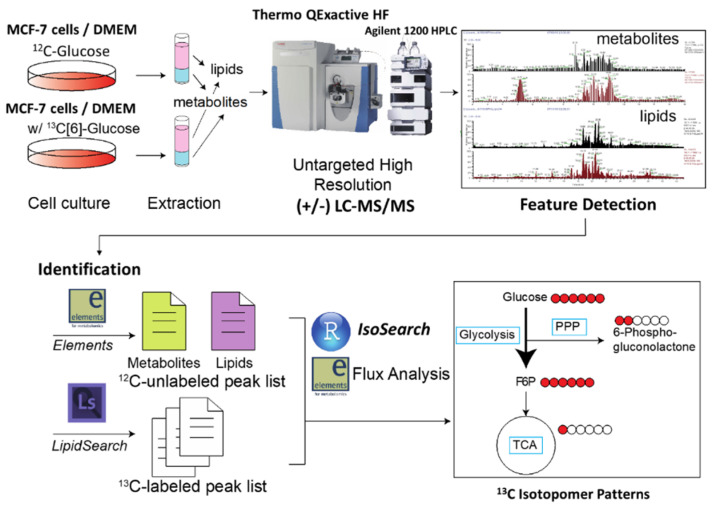
The workflow of untargeted fluxomic analysis. Two sets of cells are cultured in ^12^C-glucose or ^13^C[6]-glucose containing media for isotope labeling. Both non-polar lipids and polar metabolites were extracted using MTBE/methanol, and samples were acquired by high resolution LC-MS/MS via data-dependent acquisition (DDA) with polarity switching. The metabolites and lipids of unlabeled samples were then identified using commercial software and serve as references for labeled samples. The isotopically labeled samples were matched against the references using the in-house R script IsoSearch or the commercial software Scaffold Elements.

Here, we introduce an untargeted metabolic isotopomer tracing strategy which uses high resolution LC-MS/MS in polarity switching mode to trace the stable isotopic tracer’s (i.e., ^13^C[6]-glucose) fate and profile the results using an in-house R program named IsoSearch. As the core portion of the untargeted isotopomer tracing workflow (Figure 1), IsoSearch can take untargeted metabolomics or lipidomics results generated by high-resolution MS and deconvolute the heavy isotopic metabolite or lipid spectra, and eventually used for flux analysis. Identities of metabolites and lipids are obtained via data-dependent acquisition (DDA) and Scaffold Elements software (Proteome Software) matching against the (National Institute of Standards and Technology (NIST) 2017 and Human Metabolome Database (HMDB) spectral libraries, or by using LipidSearch [26] from the unlabeled dataset. Two sample sets (unlabeled and ^13^C or ^15^N labeled) are acquired by the untargeted metabolomic or lipidomic platform with a positive/negative polarity switch high-resolution MS. The isotopomers of the metabolites and lipids are interpreted from ^13^C isotopic labeled samples using the IsoSearch program. Using IsoSearch, every identified metabolite and lipid molecule can produce an isotopomer pattern since IsoSearch pools unlabeled samples and compares each [13] C isotopic labeled sample to the unlabeled pool. One of the core features of the IsoSearch algorithm is to screen out potential false positives using a score that incorporates both *m/z* and retention time (RT) simultaneously. We also have introduced this method as a feature in the commercial Scaffold Elements 2.1 software (http://www.proteomesoftware.com/products/elements/) which provides a user-friendly interface. As an example of the technology, we show MCF-7 human breast cancer cells after treatment with known inhibitors of central carbon metabolism and compare how universally labeled ^13^C[6]-glucose carbon atoms are tracked throughout metabolic processes. Our specific focus was concentrated on aerobic and anaerobic glycolysis, pentose phosphate pathway (PPP), tricarboxylic acid cycle (TCA), and glucose associated phosphatidylinositol 3-kinase/Akt kinase/mammalian target of rapamycin (PI3K/Akt/mTOR) signaling pathways to examine the well-known glucose involved central carbon metabolism, but also to reveal new metabolic fluxes in alternative and potentially unknown pathways.

## 2. Results

### 2.1. Development of IsoSearch Strategy-Identification and Peak Picking

Two sets of RAW untargeted high-resolution data were acquired, unlabeled (^12^C) data from control samples and ^13^C labeled datasets from various experimental cell conditions. The ^12^C-unlabeled control files were processed by Scaffold Elements 2.1 software. Using a 0.7 ID score and 0.5 MS2 score cut-off against NIST 2017 database (652,475 MS/MS spectra), more than 470 metabolites were selected. The identified metabolites were then exported as a pooled reference peak list with all related information including the *m/z* and retention time (RT). The ^12^C-unlabeled lipid. RAW files were processed similarly to obtain the pooled reference peak list with lipid *m/z* and RT information. However, instead of using Scaffold Elements, lipids were identified using Thermo LipidSearch 4.1.3 software. Lipids graded A-C and m-score ≥5.0 were accepted. MS2 data is necessary only for the identification process. Further isotope tracer analysis is performed on the MS1 level.

The ^13^C-labeled RAW files from both metabolite and lipid datasets were converted to mzXML files and MS1 peak lists were created using the R package enviPick(v1.0) (https://rdrr.io/cran/enviPick/). The parameters of enviPick() functions to generate the peak lists are listed in Table 1 and enviPick parameters used for our high resolution data from the QEaxctive Plus/HF Orbitrap was as follows: enviPickwrap (filepath.mzXML, MSlevel = c(1), dmzgap = 15, dmzdens = 4, ppm = TRUE, drtgap = 300, drtsmall = 20, drtdens = 250, drtfill = 10, drttotal = 200, minpeak = 3, recurs = 3, weight = 3, SB = 2, SN = 3, minint = 1E4, maxint = 1E7, ended = 2, ion_mode = positive/negative, progbar = FALSE).

### 2.2. IsoSearch Analysis

The ^13^C-labeled peak lists were then compared against the ^12^C-unlabeled pooled reference peak list using an in-house R program “IsoSearch”. It is important to note that only well annotated identified lipids or metabolites from the unlabeled reference samples can be used for further isotope tracer studies. The IsoSearch program performs the untargeted metabolomic/lipidomic isotope tracing analysis in two steps using multiple functions (Table 1). In the first step, IsoSearch reconstructs the ^12^C-unlabeled reference peak list by adding isotopic mass difference (^13^C/*z* = 1.0033548378/z) to each metabolite/lipid feature’s *m/z* value. Then, the ^13^C-labeled samples are searched against the reconstructed ^12^C-unlabeled reference peak list to sort out the metabolite isotopomers that have the closest *m/z* and RT. The IsoSearch program introduced a scoring algorithm to adjust the metabolite’s isotopomer accuracy and screen out the false positive identifications using the equation:

(1) $$Score=1-\sqrt{{\left(\Delta \;m/z\times \beta \right)}^{2}+\;{\left(\Delta \;RT\right)}^{2}}$$

where in the Δ *m/z* and Δ RT are the *m/z* and RT differences between isotopic labeled feature list vs. reference list, respectively. *β* (s ∙ C/g) is a unit-fixed parameter, which can justify the *m/z* and RT weight coefficient and normalize unit differences. In this article, we set *β* value to 0.1 for both the metabolite and lipid isotopomer searches. For high resolution and high mass accuracy data with Orbitrap technology, the Δ *m/z* is~1.4 ppm and Δ RT is~0.14 sec. For polar metabolites, the maximum number of possible isotopomers is 10, while the number of non-polar lipid isotopomers maxes out at 40 because of the relative number of carbon atoms in each molecule type.

The results generated by the IsoSearch:Flux_result function were summarized as a spreadsheet (.csv) whose attributes were listed in the Table 2.

The above rules and parameters for *IsoSearch* have been adopted and incorporated into Scaffold Elements metabolomics software v2.1 distributed by Proteome Software, Inc. (Portland, OR, USA), (http://www.proteomesoftware.com/products/elements/).

### 2.3. LC-MS/MS Data Preprocessing

The SRM data generated from targeted metabolomics were integrated to peak area values using MultiQuant 3.0 software (AB/SCIEX) and text output. The targeted data was acquired only to test the competency of the untargeted metabolomics IsoSearch workflow. More experimental details, including the full isotopomer datasets from both lipids and metabolites, are included in the Supplementary Materials Section.

Since we have a well established method for targeted metabolic flux analysis in our laboratory [8], we developed a methodology for unbiased and untargeted metabolic isotopomer tracing analysis suitable for any biological source whereby ^13^C or ^15^N atoms can be incorporated into the organism’s metabolism. Our goal was to utilize high resolution untargeted metabolomics and lipidomics as a way to trace the isotopomers and to discover more metabolite features. We designed the technique and software parameters using MCF-7 breast cancer cells. In order to test the power of the flux methodology for untargeted metabolomics and lipidomics data, we validated known metabolites in central carbon metabolism against targeted SRM technology [8].

## 3. Discussion

### 3.1. Principle of IsoSearch Based Metabolic/Lipid Isotopomer Tracing

Two major components of untargeted ^13^C-isotopomer tracing should be considered in the workflow; (1) discovering unbiased metabolites for flux analysis; and (2) minimizing false positive isotopomer IDs. The IsoSearch software implements untargeted heavy isotopic pattern interpretation with low false positive rate. To demonstrate the capabilities of this untargeted isotopomer-tracing platform, we used four drugs that inhibit metabolic or kinase signaling pathways in the MCF-7 breast cancer cell line, which was cultured in ^13^C[6]-glucose media, and LC-MS/MS profiled the ^13^C isotopomers at three time points after drug treatments (2 h, 16 h, and 24 h). Metabolites and lipids were extracted and run via both untargeted metabolomic and lipidomics with positive/negative polarity switching. The analysis of all metabolites and lipids as well as their associated ^13^C isotopomers was unbiased. The metabolic/lipid heavy isotopomers are the features that elute at the same time as the unlabeled metabolites with *m/z* values that increase by M + 1, M + 2, M + 3, etc. IsoSearch selects all the ^13^C isotopomers that have the identical retention time (RT) and theoretical *m/z*, and also filters off the unrelated features (i.e., the false positives). Due to the high degree of carbon incorporation of lipid isotopomers, falsely identified features occur more frequently in the lipid isotopomer profiling. It should be noted that IsoSearch is capable of accurately picking out the correct lipid isotopomers and screening out all of the false positives.

Another mission of untargeted ^13^C-fluxomics using IsoSearch technology is to identify novel metabolic and lipid features and discover ^13^C incorporation of metabolites and lipids detected in both positive and negative modes. The metabolites or lipids in ^12^C unlabeled samples were identified using Scaffold Elements (NIST 2017 MS/MS spectral library) or LipidSearch (internal lipid library), respectively. The identified metabolites or lipids from the unlabeled samples were then pooled as an identification list. The IsoSearch algorithm uses the pooled identification list as the reference for identifying and quantifying ^13^C isotopomers, and ^13^C-labeled samples are compared to the pooled metabolite/lipid reference list with a tight *m/z* and RT match. The identification of metabolite/lipid ^13^C isotopomer distribution is dependent upon the metabolite/lipid candidates that are identified via DDA from the reference samples; thus, the more molecules that are discovered in the reference samples, the more metabolites and their associated isotopomer changes can be revealed in the ^13^C-labeled samples by the untargeted approach. Since multiple LC-MS/MS runs typically result in additional small molecule identifications and increased metabolite coverage, we performed multiple unlabeled runs over various treatments to expand our identification reference pool for subsequent ^13^C isotopic pattern discovering [27,28]. For untargeted metabolic fluxomics, Scaffold Elements identified a total of 340 unique metabolites, and IsoSearch found that 241 of those metabolites were paired with at least one ^13^C isotopomer for a total of 969 associated ^13^C isotopomers (Figure S1A). In addition, LipidSearch identified 844 total unique lipid ions and 485 of them contained a total of 2369 ^13^C isotopomers (Figure S1B).

### 3.2. Untargeted Isotopic Patterns Are Consistent with the Targeted Isotopomer Detection

**Figure 2:**
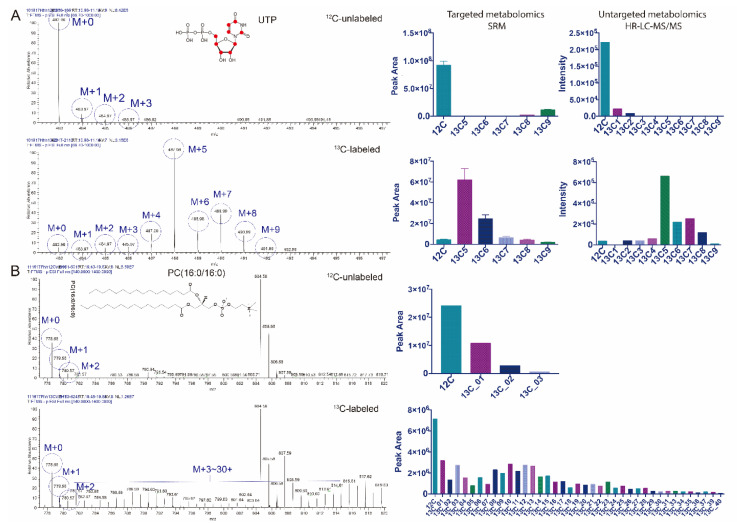
Validation of untargeted high-resolution fluxomics. (**A**) Uridine triphosphate (UTP) and its associated isotopomers were detected using both targeted and untargeted metabolomics. The abundant M+5 peak of UTP indicates the ribose ring comes from the ^13^C[6]-glucose. Both the targeted fluxomics via selected reaction monitoring (SRM) (left) and untargeted high-resolution isotopomer tracing (IsoSearch) (right) show a similar isotopomer pattern after ^13^C[6]-glucose tracing for UTP. (**B**) The phospholipid PC(16:0/16:0) and its associated isotopomers were profiled using untargeted lipid fluxomics. The overlap of lipid isotopomer peaks with high carbon numbers and other lipid peaks are distinguished using the IsoSearch strategy.

We set out to validate the results of IsoSearch software by comparing the isotopomer results of several overlapping metabolites with a well-established targeted SRM ^13^C isotopic analysis technology [8,9,10]. As an example, ^12^C-unlabeled and ^13^C-labeled uridine triphosphate (UTP) was detected using both the targeted and untargeted methods (Figure 2A). The ^13^C[6]-glucose labeled cells can flux ^13^C atoms through UTP, which accumulates in the (M+5) isotopomer peak in UTP, likely in the pentose sugar [29]. The high-resolution MS spectra detected in the untargeted metabolomic analysis interpreted by IsoSearch also indicate the same isotopic pattern as the targeted method. Several other metabolites including alanine and lactate are also detected the same isotopic patterns in both untargeted and targeted approaches (Figure S2), thus, further validating the IsoSearch algorithm. In addition, the same metabolite (S-adenosylmethionine) isotopomer profile can be obtained with both IsoSearch and Scaffold Elements 2.1 (Figure S3), which contains a ^13^C metabolic flux algorithm built from the parameters used by IsoSearch.

In addition to IsoSearch’s interpretation of the metabolite isotopomer patterns, intact lipid isotopomers can also be profiled. Most phospholipids contain a large number of carbon atoms (>30) which makes isotopomer interpretation difficult since lipids of similar size of the same class are more likely to have a similar retention time. Like metabolites, lipids are first identified using the M+0 (^12^C only) ion from a MS/MS based identification software (Thermo LipidSearch). Since lipids are larger in size than metabolites with many more carbon atoms, peak overlap can often occur. The phosphatidylcholine lipid, (PC(16:0/16:0)) (*m/z* 778.56) was detected in both ^12^C-unlabeled and ^13^C-labeled samples (Figure 2B), and co-eluted with a heavier lipid at *m/z* 804.58 [PC(16:0/18:1) + (HCOO-)]. Although the raw spectra indicate that the (M+30) to (M+40) peaks of PC(16:0/16:0) are overlapping with the (M+0) to (M+10) peaks of the heavier lipid (Figure 2B, left panel), using the score screening algorithm d, IsoSearch can differentiate the overlapping peaks from PC(16:0/16:0) isotopomers (Figure 2B, right panel).

### 3.3. Untargeted Metabolic Time-Course Analysis Reveals Drug Stimulated ^13^C[6]-Glucose Flux Alteration

Cancer signaling pathways are frequently associated with metabolic regulations [30,31,32]. In order to demonstrate the *IsoSearch* strategy and study metabolic regulation in cancer cells, the estrogen receptor (ER) positive MCF-7 breast cancer cell line, was cultured in either ^12^ C regular glucose DMEM or with the addition of universally ^13^C labeled (U-^13^C_6_) glucose DMEM to investigate the glucose metabolic flux. Four metabolism or kinase inhibitors, 2-deoxy-glucose (2DG), 6-aminonicotinamide (6AN), compound 968 (C968) and rapamycin (Rapa), were added to the cell culture media to alter the flux of glucose in MCF-7 for different purposes.

To investigate the influence of metabolic inhibitors in MCF-7 cells, 2DG was used to inhibit the production of glucose-6-phosphate and the downstream glycolysis pathway [33] (Figure S4A). 6AN was used to inhibit the pentose phosphate pathway (PPP) by blocking 6-phosphogluconate dehydrogenase [34] (Figure S4B). C968 is a glutaminase inhibitor used to block glutamine to glutamate production in the TCA cycle though it does not directly affect glucose metabolism [35,36]. Finally, Rapa was used as an mTOR inhibitor to test its effect on glucose metabolism [37]. The polar metabolite isotopomer profiles of MCF-7 breast cancer cells were analyzed by both targeted and untargeted metabolomics (Figures S5–S10). The targeted data was used as a control for some key central carbon metabolites while the untargeted data was used for discovery. As expected, 2DG induced glucose phosphorylation inhibition resulted the reduction of glycolytic flux compared to the vehicle (DMSO) and other inhibitors (Figures S5–S7). The ^13^C isotopomer incorporations of the glycolysis intermediates (fructose-6-phosphate, fructose-1,6-bisphosphate, glyceraldehyde-3-phosphate, 3-phosphoglycerate, pyruvate, etc.) were effectively reduced at the 2-h time point (Figure S5) and remained low over 24 h (Figures S6 and S7). The 6AN treatment behaved as expected by inhibiting 6-phosphogluconate dehydrogenase and the accumulation of ^13^C isotopomers of 6-phosphogluconolactone and 6-phosphogluconate over 24 h [34] (Figures S5–S7). This inhibition resulted in a decrease in ^13^C isotopomer incorporation into ribose-5-phosphate and PPP metabolism. The targeted isotopomer tracing results also showed that C968 had little effect on glucose metabolism, whereby most of the glycolytic intermediates displayed high levels of [13] C incorporation as compared to the other three metabolic inhibitors. The inhibition of mTOR kinase by Rapa decreased the ^13^C incorporation into phosphoribosyl pyrophosphate (PRPP) over 24 h (Figures S5–S7). This depletion of the PRPP ^13^C isotopomers resulted in reduced nucleic acids biosynthesis flux at 2 h and 16 h, which indicated mTOR inhibition can decrease pyrimidine and purine biosynthesis [9,38].

**Figure 3:**
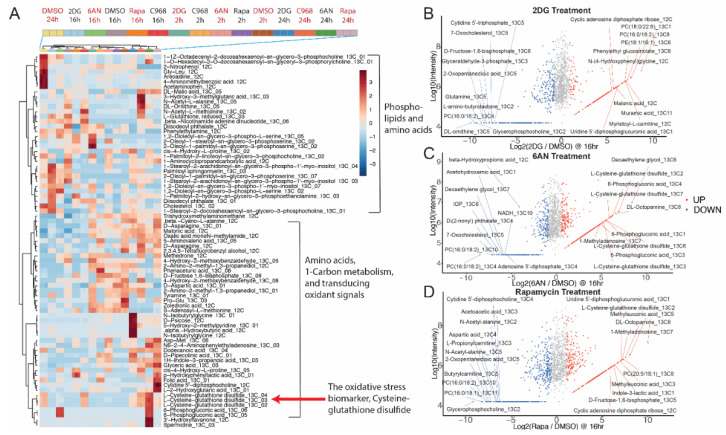
An overview of untargeted fluxomics analysis from drug treated MCF-7 breast cancer cells reveals the changes in metabolites and their associated isotopomers. (**A**) A heat map of untargeted fluxomics results displays various metabolites and their isotopomer alterations after drug treatments with 2DG, Rapa, 6AN and DMSO vehicle control. The MA scatterplot of (**B**) the top 10 increased (red) and decreased (blue) metabolites induced by 2DG at 24 h; and (**C**) the top 10 increased (red) and decreased (blue) metabolites induced by 6AN at 24 h; and (**D**) the top 10 increased (red) and decreased (blue) metabolites induced by rapamycin at 24 h.

Simultaneously, we collected untargeted metabolomic results for the time-course analysis at 2-h, 16-h, and 24-h drug treatments (Figures S8–S10). The metabolites and their associated ^13^C isotopomers revealed the same flux alterations as the targeted methods, such as the glycolysis inhibition by 2DG and PPP inhibition via 6-phosphogluconolactonate accumulation by 6AN. Moreover, untargeted metabolic ^13^C isotope tracer analysis using IsoSearch can be useful to discover novel or unexpected metabolic and lipidomic pathways which are not profiled by targeted methods. In general, the untargeted metabolomics results in MCF-7 breast cancer cells revealed that ^13^C isotopomers of some phospholipids, amino acids, 1-carbon metabolites, and signaling intermediates were highly dysregulated by the metabolic inhibitors (Figure 3A). The exceedingly increased metabolites implied metabolic isotopomers were altered by drug treatments in MCF-7 cells (Figure 3B–D). For instance, the dramatically elevated ^13^C-labeled modified amino acids (^13^C_5_-3-hydroxy-3-methylglutarate, ^13^C_5_-N-acetyl-alanine and ^13^C_5_-ornithine) in C968 treated cells indicated that ^13^C atoms fluxed to the amino acid via urea cycle [39] and amino acid catabolism [40,41] due to glutaminase inhibition; 3-hydroxy-3-methylglutarate (HMG) and N-acetyl-alanine are two amino acids–valine and alanine derivatives which can be catabolized via the urea cycle and increase ornithine ^13^C isotopomers was observed after C968 treatment; the hepatic glutaminase dysfunction can provide the urea cycle with ammonia, as previously described [42,43]. The highly accumulated fully ^13^C labeled 6-phosphogluconate by 6AN treatment was due to the inhibition of 6-phosphogluconate dehydrogenase [34] since 6-phosphogluconate links the glycolysis and PPP, and the 6AN inhibits the ^13^C atom flow from glucose-6-phosphate to pentose phosphate were observed in myelogenous leukemia and pheochromocytoma [44,45].

### 3.4. Drugs Induce Oxidative Responses in MCF-7 Breast Cancer Cells

**Figure 4:**
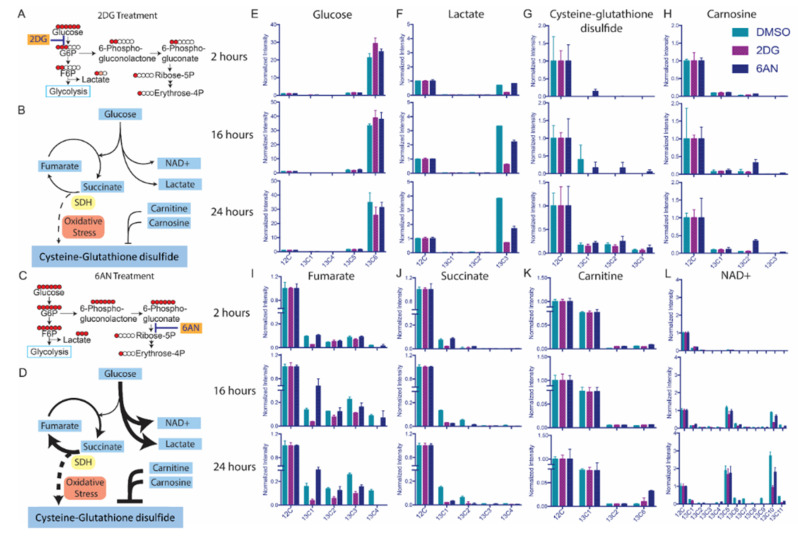
The 2DG and 6AN affect metabolic fluxes of ^13^C-glucose in MCF-7 cells differently. The bar graphs indicate the ratio of ^13^C incorporation for the isotopomers of each metabolite via IsoSearch. (**A**) 2DG inhibits the formation of glucose 6-phosphate and ^13^C flux to glycolysis. The circles represent the carbon atoms of each polar metabolite, and ^13^C-labeled carbons are filled in red. (**B**) 2DG inhibits ^13^C[6]-glucose metabolic flux and its downstream pathways. The arrow thickness represents the relative flux ratio through the metabolic pathways. (**C**) The 6AN inhibits 6-phosphogluconate dehydrogenase and ^13^C flux to PPP. (**D**) The 6AN drives the ^13^C[6]-glucose metabolic flux to aerobic glycolysis and oxidative stress. (**E**) Glucose, (**F**) Lactate, (**G**) Cysteine-glutathione disulfide, (**H**) carnosine, (**I**) fumarate, (**J**) succinate, (**K**) carnitine, (**L**) NAD+ isotopomer incorporations are altered by 2DG or 6AN at 2 h, 16 h, and 24 h.

The levels of an oxidative stress biomarker, L-cysteine-glutathione disulfide, were different in 2DG and 6AN treated MCF-7 cells (Figure 3A), which could be attributed to metabolic pathways, such as aerobic glycolysis, electron transport chain (ETC), and redox reactions [46]. 2DG inhibited glycolysis so that ^13^C labeled glucose was blocked from flowing to downstream intermediates (Figure 4A,B). It has been reported that cancer cells are more likely to catabolize glucose through the fermentation pathway and produce large quantities of lactate [47,48]. However, due to 2DG inhibition, ^13^C[6]-glucose (Figure 4E) was transported into the MCF-7 cells, but did not flux the ^13^C to lactate (Figure 4F) or TCA cycle intermediates (Figure 4I,J). Nevertheless, the untargeted fluxomics results indicated that 6AN increased the levels of ^13^C_3_-lactate (Figure 4F), ^13^C_5_-NAD + and ^13^C_10_-NAD + at the three time points (Figure 4L). Once 6AN blocked the ^13^C flux to PPP, flux to the other glucose catabolism pathways were upregulated, and ^13^C_6_-glucose was converted to fully labeled lactate with the help of NAD + in MCF-7 cells (Figure 4C,D). The 6AN also increased the flux to fumarate in the TCA cycle over 24 h (Figure 4I) while altering succinate flux differently which may indicate a succinate dehydrogenase (SDH) involved electron transport chain (ETC) disturbance (Figure 4J). SDH catalyzes the conversion of succinate to fumarate and is an important respiratory enzyme involved in redox reactions in the mitochondria [49,50]. The ^13^C isotopic distribution of succinate by 6AN treatment indicated that succinate was highly labeled at 2 h with predominantly four ^13^C atoms, but the ^13^C atoms flowed to fumarate after 16 h and 24 h. In metabolic homeostasis, the energy intake and biomass exchange in cells are maintained at a balanced rate. However, it has been reported that an altered TCA cycle network can induce reactive oxidative stress [51]. Using a DCFDA/H2DCFDA fluorescent assay (see Supplemental Materials section), ROS production in MCF-7 cells was detected at 2-h, 16-h, and 24-h time intervals after drug treatments (Figure S14). At 2 h, the ROS levels in the 6AN treated cells were dramatically increased, while they slowly dropped over the next 22 h (Figure S14A). This indicated that 6AN can stimulate ROS generation in MCF-7 cells over a short time period, but the cells later produce antioxidant chemicals to counteract intracellular ROS. The results indicated that 6AN induced a flux imbalance from succinate to fumarate that can result in the perturbation of ETC and accumulation of ROS, which was also observed in ischemic reperfusion injury in the mouse by invoking oxidative stress [52]. One of the oxidative stress biomarkers [53,54,55], the ^13^C isotopomers of L-cysteine-glutathione disulfide were increased with 6AN treatment (Figure 4G). In order to defend against intracellular ROS generation, MCF-7 cells utilized reductive species to eliminate oxidative stress and maintain the redox homeostasis. Two antioxidant compounds, carnitine and carnosine, were detected with a dramatically higher ^13^C isotopomer incorporation with 6AN treatment (Figure 4K,H). The ^13^C atoms from ^13^C[6]-glucose flowed to carnosine and carnitine at later time points (16 h and 24 h) which implied that oxidative stress defense was stimulated by the ROS.

### 3.5. Lipid Isotopomer Regulation by Rapamycin Treatment

**Figure 5:**
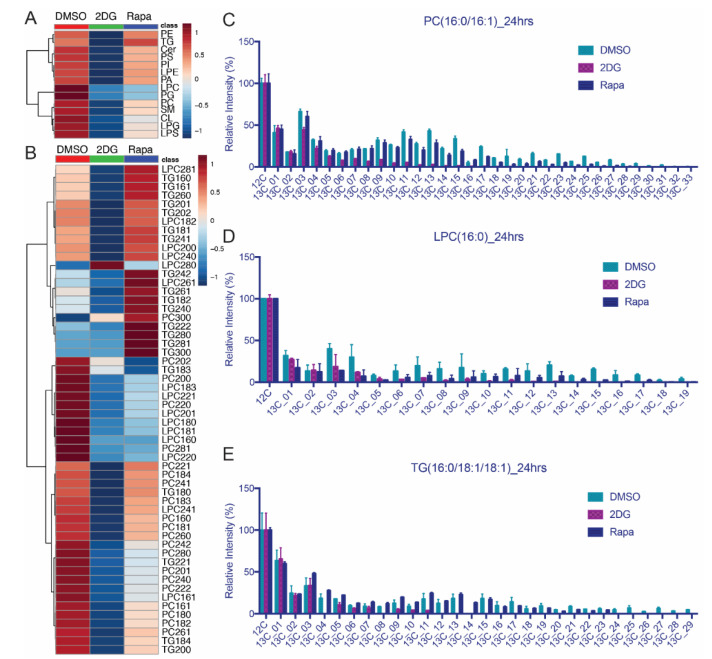
Lipid ^13^C isotopic flux changes are profiled using untargeted LC-MS/MS with polarity switching. (**A**) The 2DG and Rapa dysregulate various lipid classes compared to the control (DMSO). (**B**) The 2DG and Rapa alter the phosphatidylcholine (PC), lysophosphatidylcholine (LPC), and triglyceride (TG) lipid classes with various fatty acid chains differently. (**C**) The 2DG decreases all of the PC(16:0/16:1) isotopomer levels, but Rapa only decreases PC(16:0/16:1) flux through higher ^13^C isotopes (>M+11). (**D**) Both 2DG and Rapa down-regulated the levels of LPC(16:0) ^13^C isotopomers. (**E**) TG(16:0/18:1/18:1) isotopomer levels decreased with 2DG treatment, but increased with Rapa.

Using the ^13^C glucose isotopic tracer labeling, untargeted time-course isotopomer analysis can also reveal dynamic changes in lipid metabolism. Tracing lipid isotopomers is one of the IsoSearch applications in addition to metabolic isotopomer profiling. The untargeted lipid LC-MS/MS platform is nearly identical to the polar metabolite platform except that C_18_ reversed phase chromatography is used rather than hydrophilic interaction chromatography (HILIC). Lipids were acquired with their associated isotopomers and IsoSearch was used to profile the lipid fluxes over 2, 16, and 24-h time points (Figures S11–S13). It takes a relatively long time until ^13^C flows to lipids from ^13^C[6]-glucose; therefore, we selected 24-h treatment at to investigate how the 2DG and Rapa affect lipid flux. Previous work in our lab has shown that triglycerides (TG) accumulate to high levels with overnight Rapa treatment [26]. Various lipid classes including phospholipids, acylglycerols, sphingolipids, and ceramides and triglycerides were identified using LipidSearch identification software and their ^13^C isotopomer incorporations were profiled by IsoSearch. The common lipid class isotopomers were altered after 24-h treatment with 2DG or Rapa (Figure 5A). The IsoSearch results illustrated that most of the ^13^C carbon atoms that flowed into lipids were reduced by 2DG treatment via glycolysis inhibition [56,57] (Figure 5A,B, and Figures S8 and S9). Most phospholipid classes such as phosphatidylcholine (PC) were slightly up-regulated by Rapa treatment through 16 h and then down-regulated after 24-h while the flux of lysophosphatidylcholine (LPC) and phosphatidylglycerol (PG) were down-regulated and triglyceride (TG) was up-regulated at 24 h of treatment [26,58]. Three lipid classes (PC, LPC, and TG) were selected to represent the different regulation with Rapa. Lipids with different fatty acid chain lengths can behave differently even within the same lipid class. Using IsoSearch, the isotopomer ratio of three lipid classes (PC, LPC, and TG) were profiled based on different fatty acid chain lengths (Figure 5B). Lipids containing the same fatty acid chain lengths were selected and their associated isotopomers were summarized separately. The ratio of ^13^C isotopomer intensity (sum of ^13^C isotopomer intensities/sum of ^13^C isotopomer intensities and ^12^C isotopomer intensity) was used to calculate the ^13^C isotopic incorporation value. The reduced PC flux is likely a combination of two effects; (1) the altered short fatty acid chains such as palmitate (16:0) and stearate (18:0) and (2) down-regulated isotopomer incorporation of longer fatty acid chains including arachidate (C20:0), lignocerate (C24:0), montanate (C28:0), etc.).

Using IsoSearch, more than thirty ^13^C carbon atom isotopomers were detected. The isotopic distributions of PC(16:0/16:1), LPC(16:0), and TG(16:0/18:1/18:1) were different in vehicle, 2DG or Rapa treated cells (Figure 5C–E), but all three lipids had a relatively high (M+3) (three ^13^C atoms) isotopic peak. The (M+3) ^13^C isotope peak is derived from the lipid head group from glycerol [59,60]. In the ^13^C[6]-glucose treated experiment, the ^13^C_3_-glycerol is the product of glycerol-3-phosphate, which is generated via glycolysis. 2DG blocked glycolysis and also impeded the biosynthesis of many lipids in MCF-7 cells leading to lower levels of the (M+ 3) isotopomer in PC(16:0/16:1, LPC(16:0), and TG(16:0/18:1/18:1). Rapamycin treated cells altered (PC(16:0/16:1), LPC(16:0) and TG(16:0/18:1/18:1)) differently; the ^13^C isotope distribution pattern of PC(16:0/16:1) was similar when the isotopomers were less than (M+16), the LPC(16:0) isotopomer levels were decreased by Rapa treatment while TG(16:0/18:1/18:1) increased. The higher isotopomers (>ten ^13^C atoms) decrease with an unchanged (M+3) head group peak in PC(16:0/16:1) implying that Rapa affects the phosphatidylcholine salvage pathway where the fatty acid chains are transferred from diacylglycerol(16:0/16:1) to CDP-choline [61]. Similar to the 2DG treated cells, the isotopomers including the (M+3) head group in LPC(16:0) was decreased with Rapa treatment. LPC is a partial hydrolysis product of phosphatidylcholines, which are catalyzed by phospholipase A2. Considering the unchanged level of (M+3) head group in PC(16:0/16:1), the decrease of (M+3) in LPC(16:0) in Rapa treated cells was the effect of phosphatidylcholine hydrolysis [62,63]. Once the PC hydrolysis reaction was altered by Rapa, the LPC lipids with various fatty acid chain lengths were downregulated in MCF-7 cells. Rapa treated cells showed high intensity of TG(16:0/18:1/18:1) isotopomers, which indicated the TG storage was suppressed by mTOR expression in MCF-7 breast cancer cells. The elevation of TG lipids has also been found in Rapa treated hypertriglyceridemia patients as well as the *tsc2* mutated mouse embryonic fibroblast (MEF) cells, which indicated that mTOR suppressed TG accumulation, can also occur in some other cells and patients [26,64,65] We also attempted to incorporate triple labeled stable isotope labeling by amino acids in cell culture (SILAC) proteomics to correlate any identifiable metabolic or lipidomic enzymes to our isotope tracing results (see Supplemental Materials Section, Figures S15 and S16) for multi-omics analyses.

## 4. Materials and Methods

### 4.1. Chemicals and Reagents

Methyl tertiary-butyl ether (MTBE) used for lipid and metabolite extraction was purchased from Sigma-Aldrich. LC-MS grade water, LC-MS grade acetonitrile (ACN), and HPLC grade isopropanol (IPA) were purchased from Fisher Chemical. HPLC grade methanol (MeOH) was purchased from Pharmco-Aaper. Mobile phase buffer LC-MS grade formic acid and formic acetate were purchased from Sigma-Aldrich. Fully carbon labeled ^13^C_6_ glucose for isotopic labeling was purchased from Cambridge Isotope Laboratories. High glucose DMEM and glucose-free Dulbecco’s modified eagle medium (DMEM) were purchased from Gibco. For the drug treatments, 6-aminonicotinamide, 2-deoxyglucose, compound 968 and rapamycin were purchased from Sigma-Aldrich. A Cadenza CD-C_18_ column (150 × 2 mm) was purchased from Imtakt, and an XBridge Amide HILIC column (3.5 µm, 4.6 × 100 mm) was purchased from Waters.

### 4.2. Cell Culture

MCF-7 cells were seeded on 10-cm^2^ cell culture plates and grown in high glucose DMEM media at 37 °C overnight and reached ~30% confluency. The media was replaced by ^13^C_6_ glucose DMEM (^13^C_6_ labeled) which prepared by adding 4.5 g/L ^13^C_6_-glucose into the glucose-free DMEM or regular 4.5 g/L high glucose DMEM (^12^C-unlabeled) next day, and four drugs including 2DG, 6AN, Rapa, C968, and DMSO (vehicle) were simultaneously added to the cell culture plates, respectively. The cells were harvested at 2-h, 16-h, or 24-h after the drug treatments.

### 4.3. Lipid and Metabolite Co-Extraction

The harvested cells were extracted using the methanol/MTBE based extraction method for both metabolites and lipids according to previously published studies [26,66]. The upper phase was collected to obtain the non-polar lipids, and the middle aqueous phase was collected to obtain the polar metabolites; both phases were dried in a SpeedVac at room temperature separately. Polar metabolites and non-polar lipids were stored at −80 °C until mass spectrometry analysis [26,66]. Metabolites were reconstituted in 20 µL of water and lipids were reconstituted in 35 µL of MeOH/IPA (*v:v* 50:50) just prior to LC-MS/MS analyses.

### 4.4. LC-MS/MS Based Metabolomic and Lipidomic Analyses

The reconstituted polar metabolite samples split into two equal parts for either targeted or untargeted metabolomics analysis. For targeted metabolomics, 5 µL sample was injected into the 5500 or 6500 QTRAP hybrid triple quadrupole MS (AB/SCIEX) using amide HILIC chromatography and acquired via SRM with a positive/negative ion-switching targeted analysis with 300 unlabeled targets [6,8]. For untargeted metabolomics and lipidomics, the same volume of sample was injected into a high resolution QExactive HF Orbitrap MS (Thermo Fisher Scientific) with positive/negative switching. The source voltages were 4.25 kV in both modes, the source heater temperature was 300 °C and the capillary temperature was set to 250 °C. The resolution was set to 70,000 in MS1 mode and 35,000 in MS2 mode. The top eight ions in each mode were selected for MS2 via data-dependent acquisition (DDA). In MS2 mode, an automatic gain control (AGC) target of 1e6 and a max IT of 85 msec was used. In MS1 mode, the AGC was 5e5 and IT was set to 65 msec. The untargeted metabolomics method adopted the same HPLC conditions as previously published targeted metabolomics, using amide HILIC XBridge (Waters) column with a gradient of 85% B to 2% B over 15 min. A buffer (pH = 9.0: 95% (vol/vol) water, 5% (vol/vol) acetonitrile, 20 mM ammonium hydroxide, 20 mM ammonium acetate); B buffer (100% acetonitrile) via DDA [66]. The 5 µL of the reconstituted lipid samples were injected onto the QExactive Plus LC-MS/MS system using polarity switching / DDA mode with C_18_ reversed phase chromatography [26,66] as previously published with a gradient of 32% B to 97% B over 25 min. A buffer (pH = 3.5: 39.9% (*v/v*) water, 60% (*v/v*) acetonitrile, 10 mM ammonium formate, 0.1% formic acid); B buffer (89.9% (*v/v*) isopropanol, 10.0% (*v/v*) acetonitrile, 10 mM ammonium formate, 0.1% formic acid). The untargeted lipidomics MS acquisition parameters were the same as above for untargeted metabolomics. We found that amide HILIC was the most robust and reproducible chromatographic media for polar small molecule metabolites, while intact non-polar lipids were best separated and detected using C_18_ reversed phase chromatography columns [6,8,26].

## 5. Conclusions

Isotopic tracer flux analyses typically report the detection of molecules present in well-known metabolic pathways. In this report, we have described a strategy to interpret metabolite and lipid isotopomers of polar metabolites and non-polar lipids identified from untargeted high resolution LC-MS/MS analyses, and subsequently re-identified and quantified in ^13^C isotopically labeled samples. The IsoSearch, strategy profiles the distribution of each identified molecule’s isotope profile, and providing information for subsequent polar metabolic flux modeling. We also applied our technique to a set of non-polar lipids to reveal lipid class regulation, fatty acid chain composition changes, and individual lipid isotopic distribution alterations. This R script derived isotope labeling strategy is also released as a user-friendly component of the commercial software Scaffold Elements versions 2.0 and later (Proteome Software) and requires high resolution and high mass accuracy MS and MS/MS data. The ability to perform isotope tracer analyses using polarity switching has the added benefits of conserving precious sample while also improving sample throughput and a longer chromatographic run can compensate for any potential data loss due to MS acquisition cycling. In general, IsoSearch provides metabolite/lipid isotopomer tracing capabilities to unbiased untargeted metabolomics and lipidomics to facilitate the large-scale global exploration of isotopic fluxomics from any biological source, whereby metabolites and lipids can be extracted and profiled by LC-MS/MS. Prior to isotope tracer analysis, our LC-MS/MS polarity switching platform can identify and annotate ~300–450 metabolites and ~ 800–1500 lipids for many biological sample types. The ability to trace isotopomers from any identified molecule is a unique feature of IsoSearch (and Scaffold Elements). In addition, only molecules that have been confidently identified and annotated based on MS/MS data can be further used for IsoSearch isotopomer profile tracing.

## Figures and Tables

**Table 1:** List of IsoSearch() functions.

**Function**	**Parameters**	**Description**
Flux_result()	input_negative, input_positive, referInput, score = 0.61 (default)	main function to generate the final result of the flux analysis
ref_13C_neg()	refInput, refOutput	create the reference list using negative mode unlabeled samples (^12^C)
ref_13C_pos()	refInput, refOutput	create the reference list using positive mode unlabeled samples (^12^C)
msMatch()	mzFile, refile	searching process
resultWrap_neg()	inputFile_neg, refInput	wrapping the flux ^13^C-labeling results of negative ion mode samples
resultWrap_pos()	inputFile_pos, refInput	wrapping the flux ^13^C-labeling results of positive ion mode samples
Sgrade()	inputResult	scoring function using **Equation (1)** to calculate the filtering scores
refLibPos_ls()	refInput	preserved function for *lipidSearch*
refLibNeg_ls()	refInput	preserved function for *lipidSearch*
refLibPos_ele()	refInput	preserved function for *Elements*
refLibNeg_ele()	refInput	preserved function for *Elements*

IsoSearch program built-in functions. IsoSearch() uses the ^13^C-labeling peak lists and ^12^C-unlabeled reference list for flux search processing.

**Table 2:** Description of IsoSearch() output table (.csv).

**Column Heading**	**Description**
mz1	mass to charge ratio of the searched file
rt1	Retention time value of the data file
Intensity	feature peak intensity
mz2	*m/z* value of the ^13^C-labeled experimental file
rt2	retention time value of the data file
Metabolite/Lipid	name of the feature
fattyAcid, lipidClass, lipidForm	fatty acid chain, lipid class and chemical formula of the lipid (lipid fluxomics only)
Accession	accession number assigned by the database
Theoretical_mz	isotopomer theoretical *m/z* value
Adduct	adduct ion (+H, −H, etc.)
Charge (z)	ion charge mode
Annotation	isotopomer notation (M+1, M+2, M+3, etc.)
Score	score used for feature screening
Δmz_ppm	the difference between experimental and theoretical *m/z* in ppm
Grades	Quality associated with the score where A is best

The fluxomics results generated by IsoSearch() are presented in a spreadsheet with headings.

## Supplementary Materials

The following are available online at https://www.mdpi.com/2409-9279/3/3/54/s1: Figures S1–S17, ROS detection assay and SILAC methodology and multi-omics results. Metabolite and Lipid results files have been published on the Zenodo data repository, 10.5281/zenodo.3908617.
