# Supplementary material for: IsoSearch: An Untargeted and Unbiased Metabolite and Lipid Isotopomer Tracing Strategy from HR-LC-MS/MS Datasets
Source: Methods Protoc. 2020 Jul 30;3(3):54. doi: 10.3390/mps3030054 (PMC7563207; doi:10.3390/mps3030054)
Supplement: Supplementary file 1 [file mps-03-00054-s001.pdf]

methods and protocols

MDPI

Supplementary Information

IsoSearch: An untargeted and unbiased metabolite and lipid isotopomer tracing strategy from HR-LC-MS/MS datasets

He Huang [1,2,3], Min Yuan [1], Phillip Seitzer [4,5], Susan Ludwigsen [4] and John M. Asara [1,2,*]

1 Division of Signal Transduction, Beth Israel Deaconess Medical Center, Boston, MA 02115, USA
2 Department of Medicine, Harvard Medical School, Boston, MA 02115, USA
3 Institute of Metabolism and Integrative Biology, Fudan University, Shanghai 200438, China
4 Proteome Software, Inc., Portland, OR 97219, USA
5 Calico Life Sciences, San Francisco, CA 94080, USA
* Correspondence: jasara@bidmc.harvard.edu; Tel.: +1-617-735-2651

Reactive oxygen species (ROS) detection

A DCFDA/H2DCFDA cellular ROS assay kit was purchased from Abcam. The arginine (R) and lysine (K)-free DMEM and L-arginine (R0), L-lysine (K0), ^{13}C[6]-arginine (R6), 4,4,5,5-D$_4$-lysine (K4), ^{13}C[6],^{15}N[4]-arginine (R10), and ^{13}C[6],^{15}N[2]-lysine (K8) were purchased from Cambridge Isotope Laboratories for SILAC experiments. Dialyzed fetal bovine serum (FBS) was purchased from Thermo Fisher Scientific. MS grade trypsin/Lys-C mix was purchased from Promega.

MCF-7 cells were seeded on a 96-well plate overnight, followed by 2DG, 6AN, rapamycin, compound 968, or DMSO (vehicle) treatment. After 2-hour, 16-hour or 24-hour treatments, the DCFDA fluorescence reagent was added to each well and the cells were incubated for 40 min at 37°C. The incubated 96-well plate was then measured using a fluorescent plate reader at Ex485/Em535 nm wavelength. The fluorescent intensities of all the wells were recorded as the general ROS levels produced by the MCF-7 cells.

Stable isotope labeling by amino acids in cell culture (SILAC) proteomics analysis

Three types of SILAC media were prepared by adding Arg0/Lys0, Arg6/Lys4, or Arg10/Lys8 into arginine/lysine-free DMEM to form light, medium, or heavy SILAC cell culture media. The MCF-7 cells cultured separately in the SILAC media for at least 6 cell cycles until more than 98% intracellular proteins were labeled by the stable isotopes. The light (R0/K0), medium (R6/K4), or heavy (R10/K8) isotopic labeled cells were then treated with DMSO (vehicle), 6AN, or rapamycin, respectively. The drug treated cells were lysed by radioimmunoprecipitation assay (RIPA) buffer and all the proteins were collected in 1.5 mL microcentrifuge tubes. The light, medium and heavy labeled proteins were quantified using a Nanodrop and mixed 1: 1: 1 (*w:w:w*) as a pooled protein solution. The pooled proteins were then boiled for 10 min, and 400 µg of denatured proteins was loaded into each 10% Tris-Glycine gel well for gel electrophoresis. The gel-fractionated proteins were dyed using Coomassie blue solution for one hour, and the whole gel lanes were each cut into 12 equal gel pieces. Each gel piece was digested by trypsin/Lys-C overnight at 37°C/pH=8.3, and then extracted peptides were concentrated to 10 µL. The trypsin digested proteins were acquired by a QExactive HF hybrid quadrupole-Orbitrap mass spectrometer (Thermo Scientific) and analyzed by the MaxQuant 1.6 software for protein identification and SILAC quantification[26].

MaxQuant 1.6 is an open source program acquired from https://www.maxquant.org/ (Max-Planck Institute of Biochemistry) used for SILAC protein quantification[27]. PANTHER 14.1 is an open source program for proteomic GO analysis https://www.pantherdb.org.

Multi-omics correlations between metabolite and lipid fluxomics and proteomics

Multi-omics is a powerful tool in systems biology studies that typically takes two or three high-throughput analytical methods to interpret the whole biological system[74,75]. Our lab has utilized multi-omics in a number of different studies using a combination of lipidomics, phosphoproteomics and metabolomics from studies ranging from multiple myeloma cancer cells, mouse breast tumors and equine urine[28,76,77]. The utilization of metabolite/lipid fluxomics incorporated with the other members of "omics" family help us understand the kinetic changes of metabolites or lipids in the biological system. Multi-omics integrated with metabolic flux analysis has been widely used in microbiological studies recently[78–80]. Since metabolite and lipid fluxes are strongly related to intracellular enzyme expression, we used a bottom-up proteomics method, stable isotope labeling by amino acids in cell culture (SILAC)[81], with which 1915 proteins were identified in drug treated MCF-7 cells, and compared these results to our metabolic and lipid fluxomics data.

The MCF-7 cells were unlabeled Arg0/Lys0 (light) or labeled Arg6/Lys4 (medium), or Arg10/Lys8 (heavy) SILAC media and then treated with DMSO (vehicle), 6AN or rapamycin respectively for 24 hours. The proteomics results revealed that 6AN and rapamycin dysregulated intracellular proteins (Figure S17A). Of the top 75 dysregulated proteins, both 6AN and rapamycin induced down-regulation in 17 proteins while rapamycin down-regulated an additional 44 proteins associated to cellular processes (27%) and metabolic processes (43%) (Figure S15A). The 10 proteins up-regulated by 6AN and 8 proteins up-regulated by rapamycin were related to cellular processes (39%), metabolic processes (31%) and biological regulation (13%) (Figure S15B). We then selected the 25 most up-regulated and down-regulated proteins in MCF-7 cells treated by 6AN (Figure S17B) or rapamycin (Figure S17C) at the 24-hour treatment time. 6AN regulated 25 metabolic process related proteins in which 18 were down-regulated (Figure S15C) and 14 were up-regulated (Figure S15D). Of the most-changed metabolic process proteins, four enzymes (SUCLG1, *Succinyl-CoA ligase subunit alpha*, SUCLG2, *Succinyl-CoA ligase subunit beta*, SDHA *Succinate dehydrogenase complex, subunit A* and FH *fumarate hydratase*) catalyze the TCA intermediates – succinate and fumarate metabolism. As we observed from the previous untargeted metabolic fluxomic results (Figure 2D), oxidative stress occurs from a 6AN induced imbalance in succinate/fumarate flux[63]. Two enzymes, PCK2 (phosphoenolpyruvate carboxykinase 2) and ASNS (asparagine synthetase) were up-regulated in 6AN treated MCF-7 cells (Figure S17B). PCK2 converts a TCA intermediate oxaloacetate to phosphoenolpyruvate, and ASNS converts aspartate to asparagine which can produce glutamate from glutamine simultaneously. This indicated that 6AN induced TCA metabolism to other metabolic pathways, a reprogramming effect. Using a ^{14}C tracing technique, Hothersall et al. found a similar phenomenon that 6AN altered the glycolytic route via glutamate-gamma-aminobutyrate pathway to the TCA cycle in rat brain[82], which could also explain the up-regulated TCA intermediate flux and down-regulated glycolysis flux in 6AN treated MCF-7 cells (Figure 3D).

We then connected central carbon metabolic enzymes from the proteomic data to the correlated metabolic flux pathways and found the enzymatic alterations mostly occurred at TCA energy generation and PPP metabolism in 6AN treated MCF-7 cells (Figure 3D). The downregulated PGLS (6-phosphogluconolactonase) resulted in decreased PPP intermediate fluxes which also linked to purine and pyrimidine metabolism. Depletion of ^{13}C-incorporation into nucleotides (adenine, cytosine, and orotidine) was observed at 24-hour (Figure S10). Nucleotide metabolism inhibited by the 6AN treatment was also found in CHO cells[83] and rat embryos[84] in 1980s. In addition, the 6AN increased an oxidative stress response protein, SOD2[85,86], which indicated that 6AN stimulated oxidative stress response on both the metabolite and protein level.

By inhibiting the activation of mTORC1, rapamycin perturbed the downstream signaling factors; SERBO-1c[66,87,88] and FASN[89,90] activities at 24-hour (Figure S17E). Of the top 25 rapamycin down-regulated proteins, nine of them linked to metabolic processes and 9 of them linked to cellular processes (Figure S15E); while rapamycin upregulated 16 metabolic process proteins and 10 cellular process ones (Figure S15F). Of the most down-regulated metabolic enzymes, MKI67 is a biomarker of cell proliferation[91] and DLD (Dihydrolipoamide dehydrogenase) plays a vital role in energy metabolism which can catalyze the oxidation of NADH to NAD^+, synthesis of acetyl-CoA, and act as

an antioxidant in the mitochondria[92–94]. This indicated a mechanism by which rapamycin can inhibit cell proliferation, energy generation, lipogenesis (Figure S13), and oxidative stress protection (Figure S14). Similarly to 6AN, the rapamycin greatly increased three TCA metabolism enzymes: FH, OGDH and PCK2 (Figure S17C). In addition to TCA metabolism, rapamycin also affected RNA signaling by promoting the levels of NSUN2 (NOP2/Sun domain family, member 2), SNRNP70 (Small Nuclear Ribonucleoprotein U1 Subunit 70), DCPS (Decapping Enzyme), and LRRFIP1 (Leucine-rich repeat flightless-interacting protein 1) and demoting the levels of two RNA binding proteins (BUB3 and ATXN2L). The up-regulated protein, an adipogenesis factor, ADIRF[95] drew our attention considering that lipid alterations were detected in rapamycin treated cells (Figure 4). Adipogenesis usually accompanies lipid droplet formation and triglyceride (TG) accumulation[96,97]. Thus, the promoted ADIRF implied the increased formation of TG would occur in rapamycin treated MCF-7 cells, which was observed in the former lipid fluxomic experiments (Figure 4A and 4E). Similarly, Yoon et al. found the enhanced adipogenesis in mTOR knockdown preadipocytes suggested the mTOR can suppress the adipogenesis[98]. This multi-omics fluxomics approach can provide another perspective to unravel the mystery of cancer signaling and metabolic disorders in a variety of human diseases.

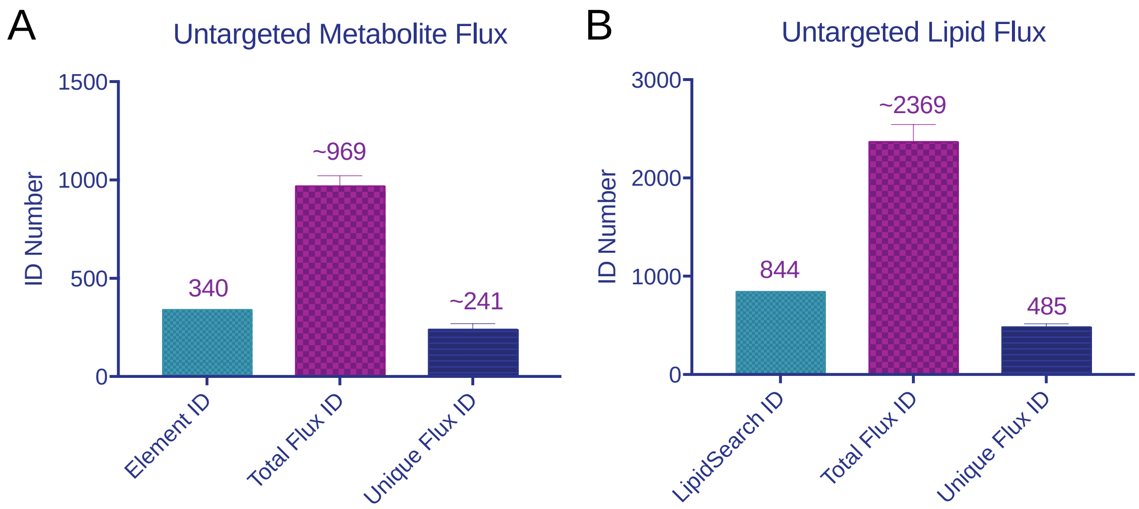

Figure S1. Identification number of untargeted metabolomics and lipidomics and associated fluxomics by high resolution LC-MS/MS. **(A)** 340 total unique metabolites were identified by *Elements* using an ID Score > 0.7 and MS2 score > 0.5; and *IsoSearch* found that 241 metabolites (unique flux ID) were ^{13}C labeled with a total of 969 ^{13}C isotopomers (total flux ID). **(B)** 844 unique lipids were identified by *LipidSearch* using > grade D and mScore > 5.0 criteria; and *IsoSearch* found 485 lipids (unique flux ID) with 2,369 ^{13}C associated isotopomers (total flux ID).

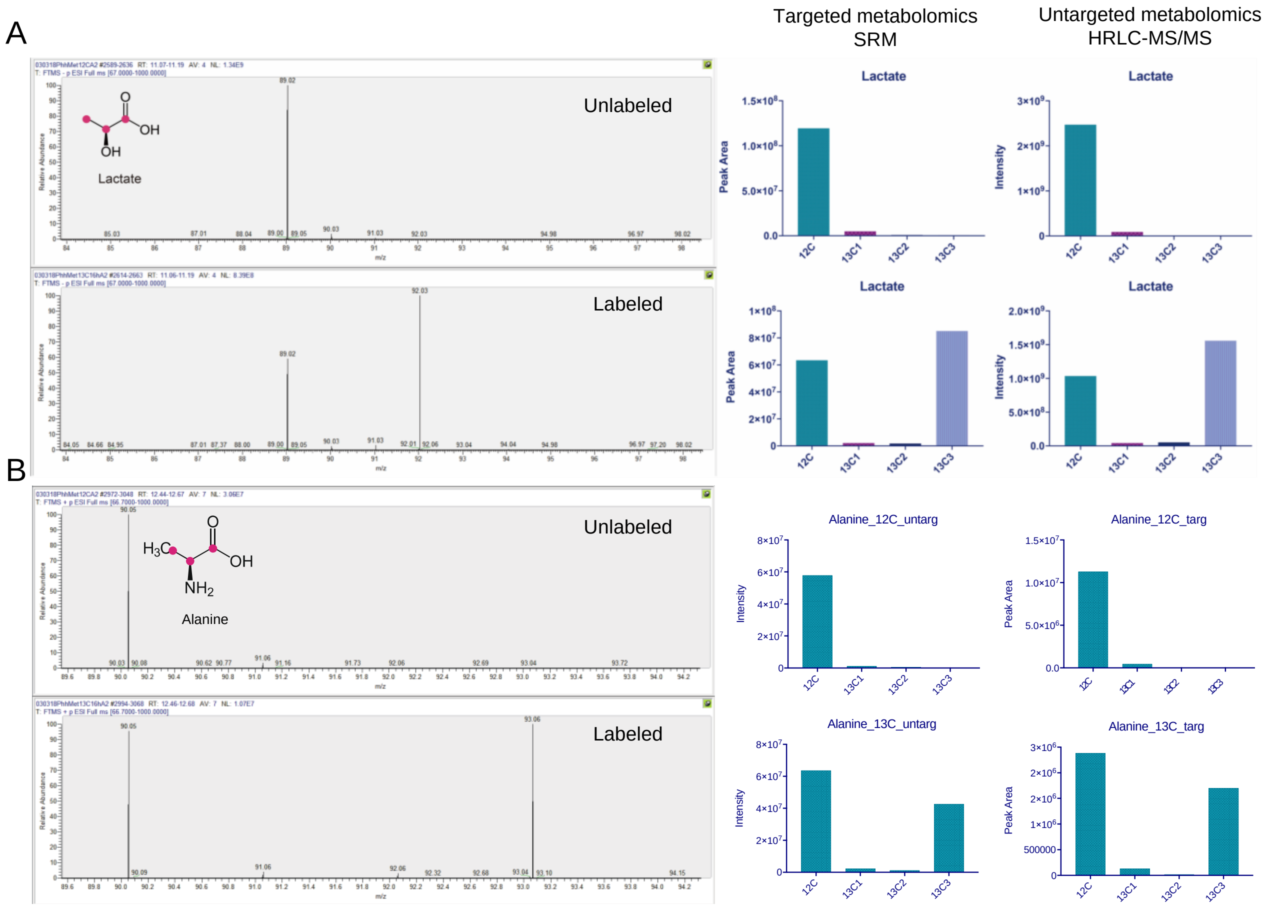

Figure S2. Lactate and alanine are detected in both targeted and untargeted metabolomics for validation of the *IsoSearch* platform. **(A)** Unlabeled or ^{13}C-glucose labeled lactate is detected (M+0: *m/z* 89.02, M+1: *m/z* 90.03, M+2: *m/z* 91.03, M+3: *m/z* 92.03) is detected by both SRM targeted and untargeted HR-LC-MS/MS. **(B)** The same isotopic pattern of unlabeled or ^{13}C-labeled alanine is detected (M+0: *m/z* 90.05, M+1: *m/z* 91.06, M+2: *m/z* 92.06, M+3: *m/z* 93.06) in both targeted (SRM) and untargeted modes (LC-MS/MS and *IsoSearch*).

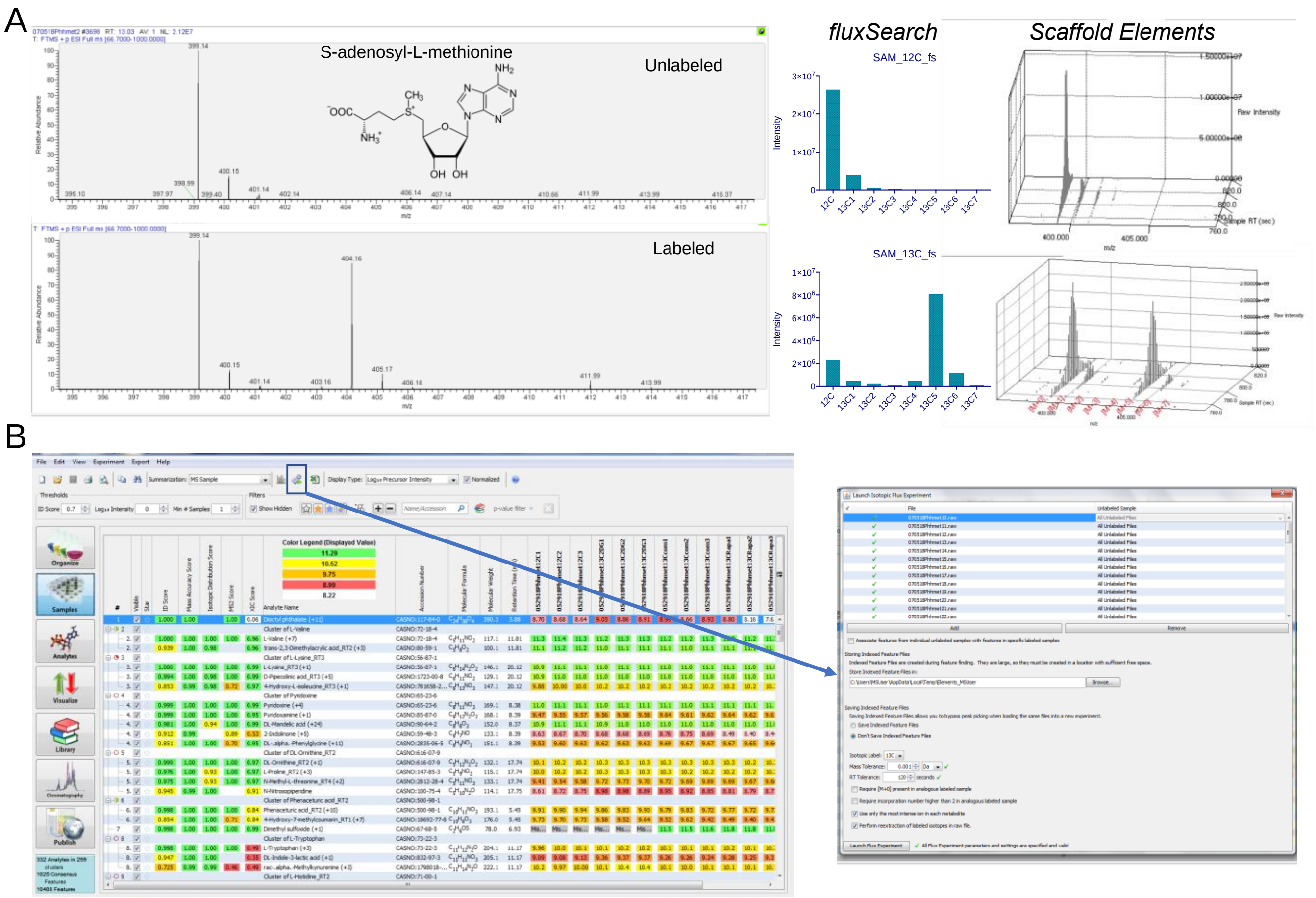

Figure S3. The isotopomer pattern of S-adenosyl-L-methionine (SAM) is identified by both *IsoSearch* program and *Elements* software which adopted the *IsoSearch* algorithm for flux analysis functionality. **(A)** The M+5 (*m*/*z* 404.16), M+6 (*m*/*z* 405.17), and M+7 (*m*/*z* 406.16) isotopomers of SAM are increased in the ^{13}C-glucose labeled sample. **(B)** The Graphical User Interface of *Elements* which incorporates *IsoSearch* functionality. .

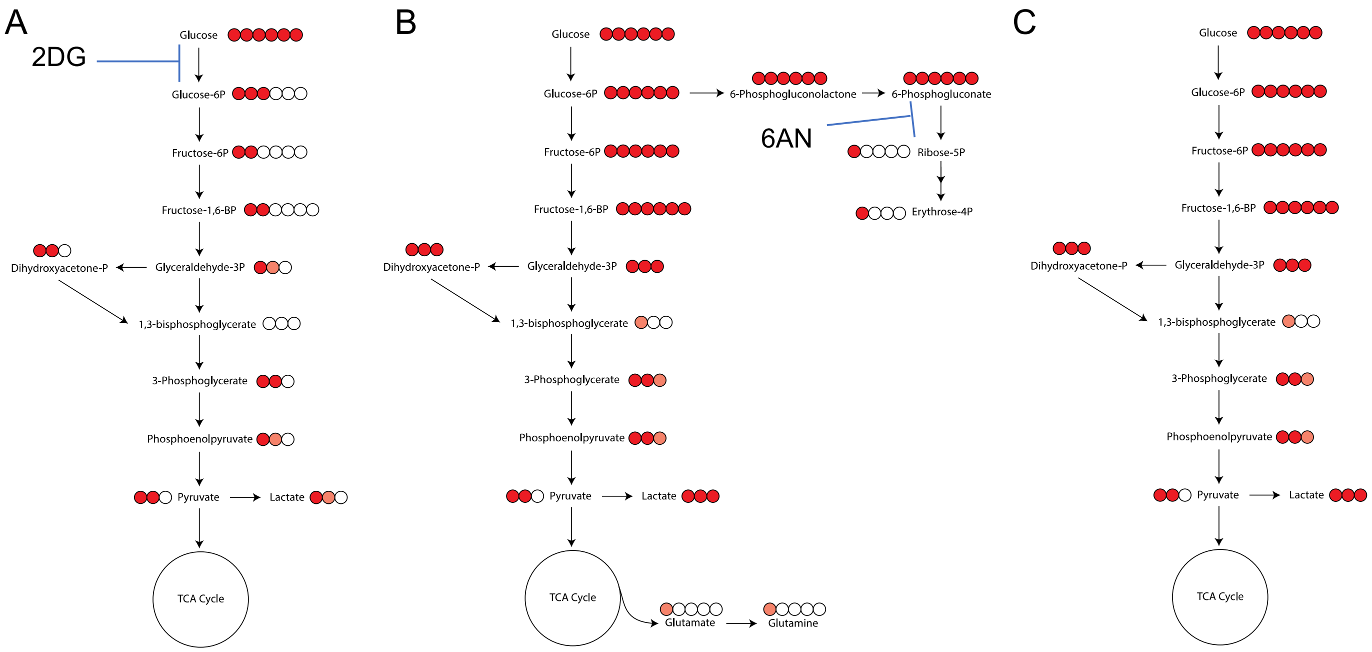

Figure S4. The ^{13}C-isotopomer distribution of glycolysis metabolism of **(A)** 2DG; **(B)** 6AN; and **(C)** DMSO treated MCF-7 cells. The circles represent the carbon atoms of each molecule and the ^{13}C-labeled carbons are filled in red color.

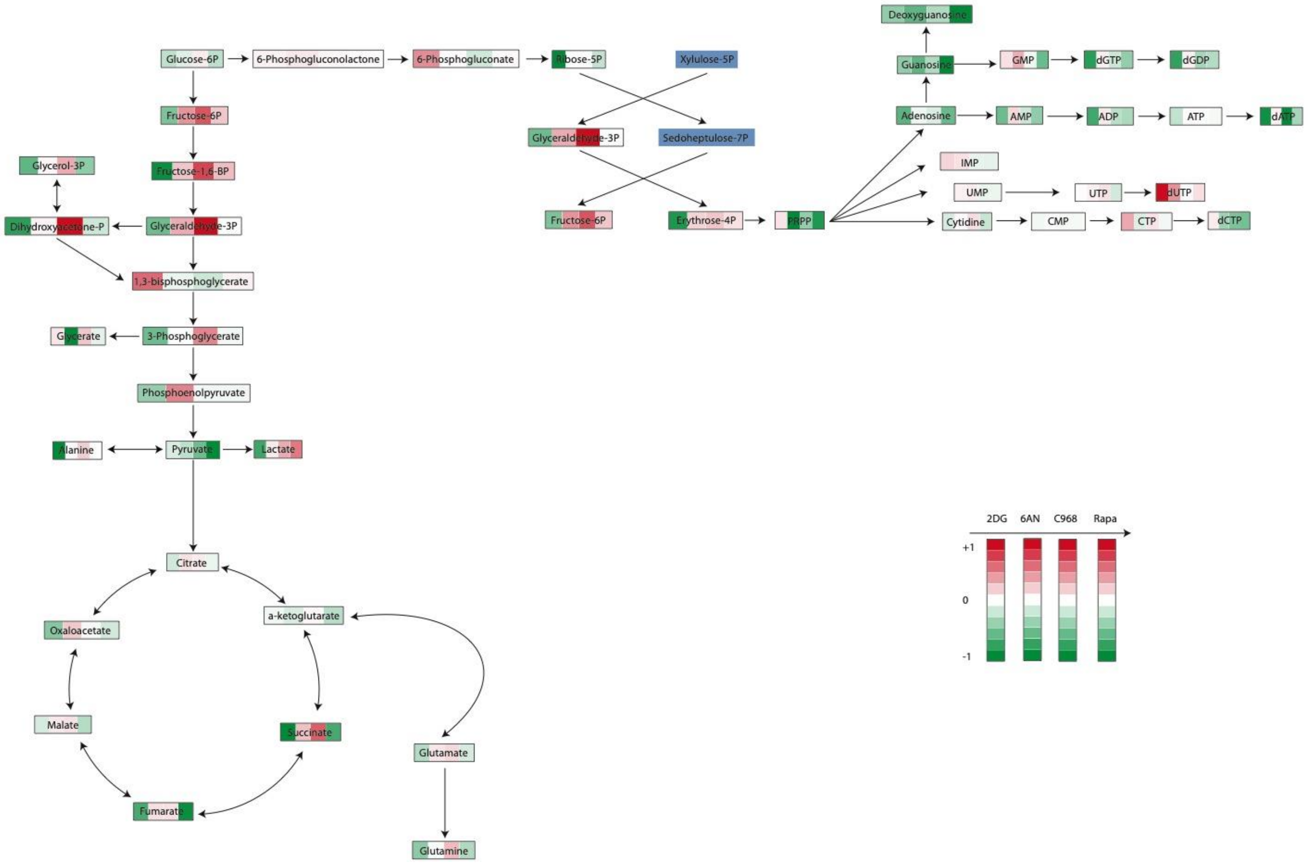

Figure S5. The metabolic flux pathway connection of 2DG, 6AN, C968 and Rapamycin treatment of MCF-7 cells at 2-hour drug treatments profiled by targeted metabolomics. The metabolites are color coded based on the ^{13}C-flux ratio calculated using the *IsoSearch* formula ($flux\ ratio_{13C} = \frac{(\sum Intensity_{13C}/\sum Intensity_{12C})_{drug\ treated}}{(\sum Intensity_{13C}/\sum Intensity_{12C})_{Vehicle}}$). The increased flux is represented in *red* color, and decreased flux is represented in *green* color. 2-DG inhibits glucose to glucose-6P, 6-AN inhibits 6-phosphogluconate to ribose-5P, C968 inhibits glutamine to glutamate and Rapa inhibits mTOR.

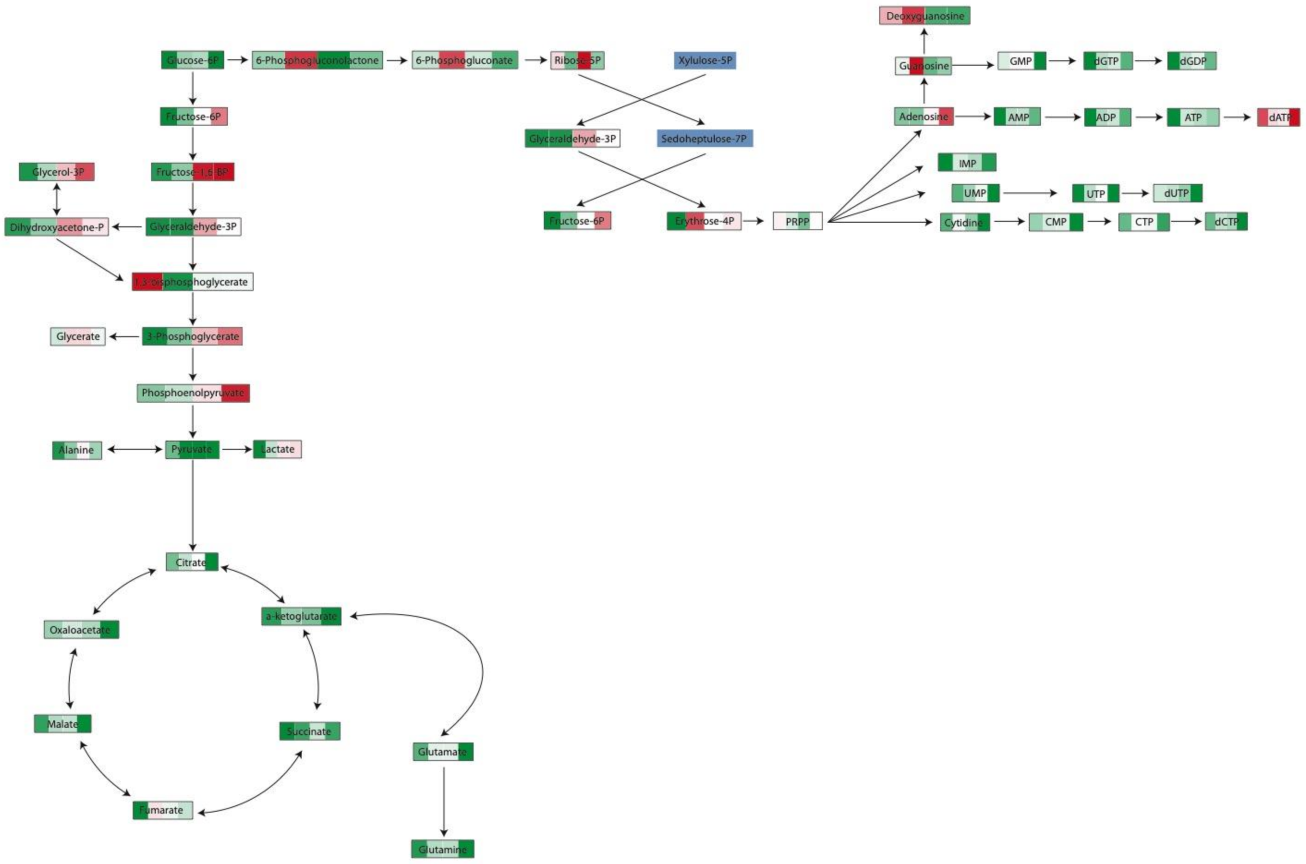

Figure S6. The metabolic flux pathway connection of 2DG, 6AN, C968 and Rapamycin treatment at 16 hours profiled by the targeted metabolomics. The metabolites are color coded based on the ^{13}C-flux ratio which calculated using formula ($flux\ ratio_{13C} = \frac{(\sum Intensity_{13C}/\sum Intensity_{12C})_{drug\ treated}}{(\sum Intensity_{13C}/\sum Intensity_{12C})_{Vehicle}}$). The increased flux is represented in *red* color, and decreased flux is represented in *green* color. 2-DG inhibits glucose to glucose-6P, 6-AN inhibits 6-phosphogluconate to ribose-5P, C968 inhibits glutamine to glutamate and Rapa inhibits mTOR.

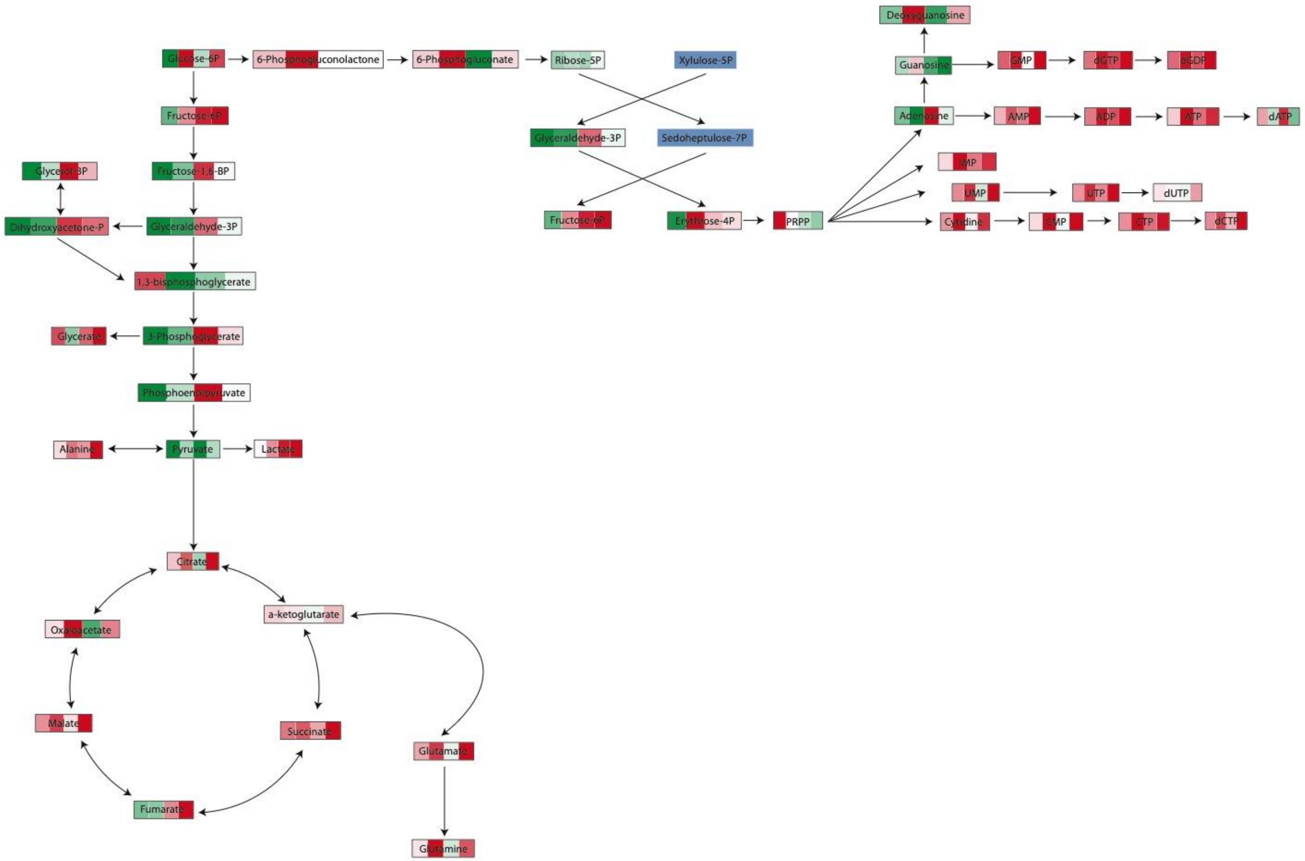

Figure S7. The metabolic flux pathway connection of 2DG, 6AN, C968 and Rapamycin treatment at 24 hours profiled by the targeted metabolomics. The metabolites are color coded based on the ^{13}C-flux ratio which calculated using formula ($flux\ ratio_{13C} = \frac{(\sum Intensity_{13C}/\sum Intensity_{12C})_{drug\ treated}}{(\sum Intensity_{13C}/\sum Intensity_{12C})_{Vehicle}}$). The increased flux is represented in *red* color, and decreased flux is represented in *green* color. 2-DG inhibits glucose to glucose-6P, 6-AN inhibits 6-phosphogluconate to ribose-5P, C968 inhibits glutamine to glutamate and Rapa inhibits mTOR.

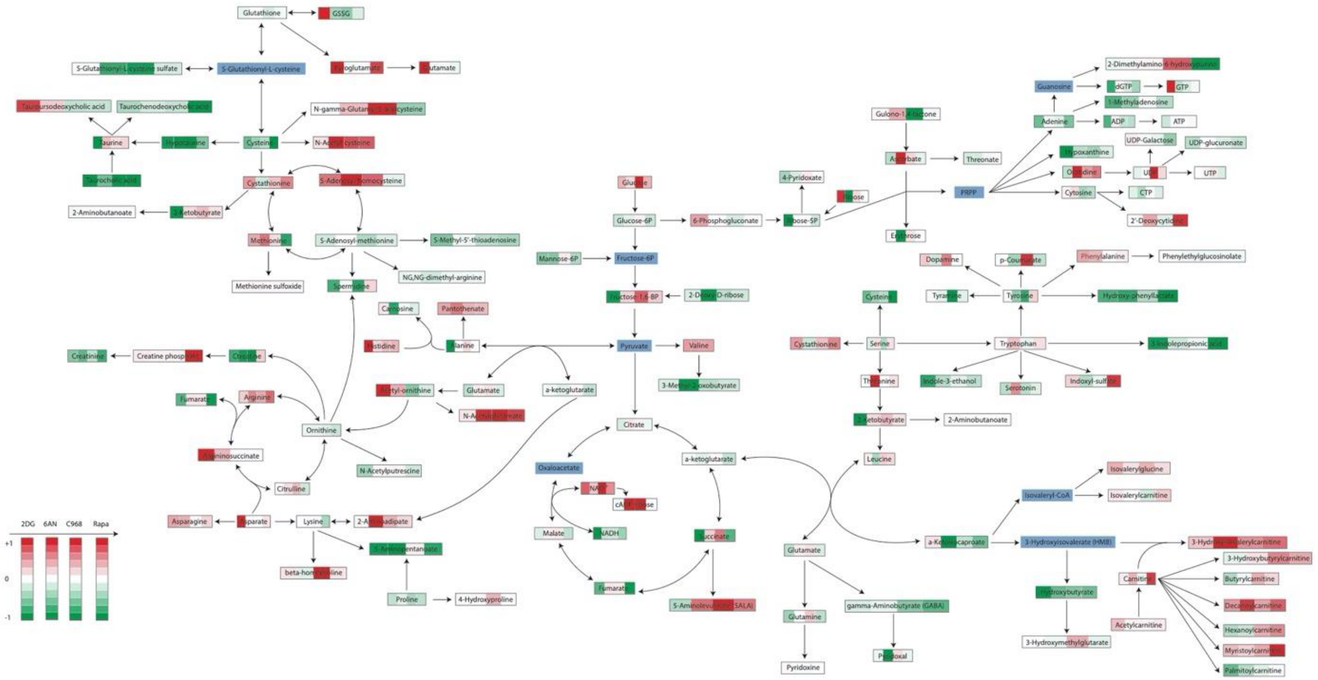

Figure S8. The metabolic flux pathway connection of 2DG, 6AN, C968 and Rapamycin treatment at 2-hour profiled by the HR-LC-MS/MS untargeted metabolomics. The metabolites are color coded based on the ^{13}C-flux ratio which calculated using formula ($flux\ ratio_{13C} = \frac{(\sum Intensity_{13C}/\sum Intensity_{12C})_{drug\ treated}}{(\sum Intensity_{13C}/\sum Intensity_{12C})_{Vehicle}}$). The increased flux is represented in *red* color, and decreased flux is represented in *green* color; and undetected metabolites are colored in *blue*.

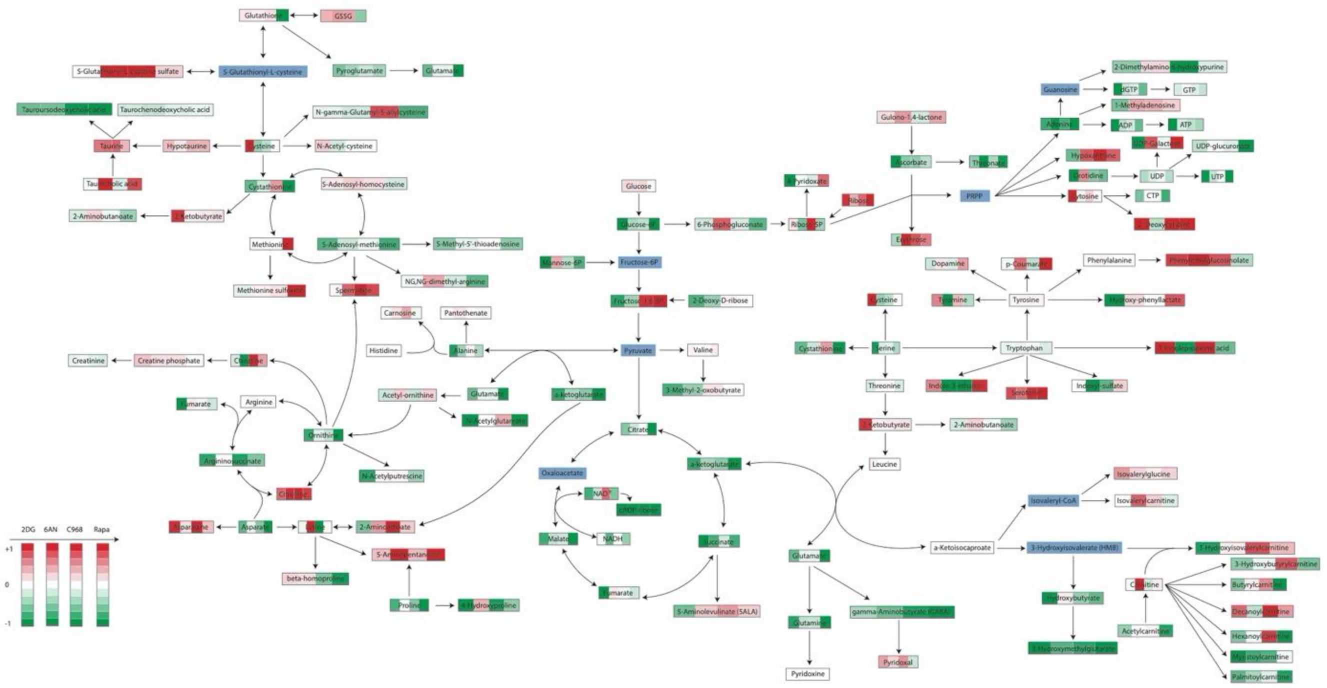

Figure S9. The metabolic flux pathway connection of 2DG, 6AN, C968 and Rapamycin treatment at 16-hour profiled by the HR-LC-MS/MS untargeted metabolomics. The metabolites are color coded based on the ^{13}C-flux ratio which calculated using formula ($flux\ ratio_{13C} = \frac{(\sum Intensity_{13C}/\sum Intensity_{12C})_{drug\ treated}}{(\sum Intensity_{13C}/\sum Intensity_{12C})_{Vehicle}}$). The increased flux is represented in *red* color, and decreased flux is represented in *green* color; and undetected metabolites are colored in *blue*.

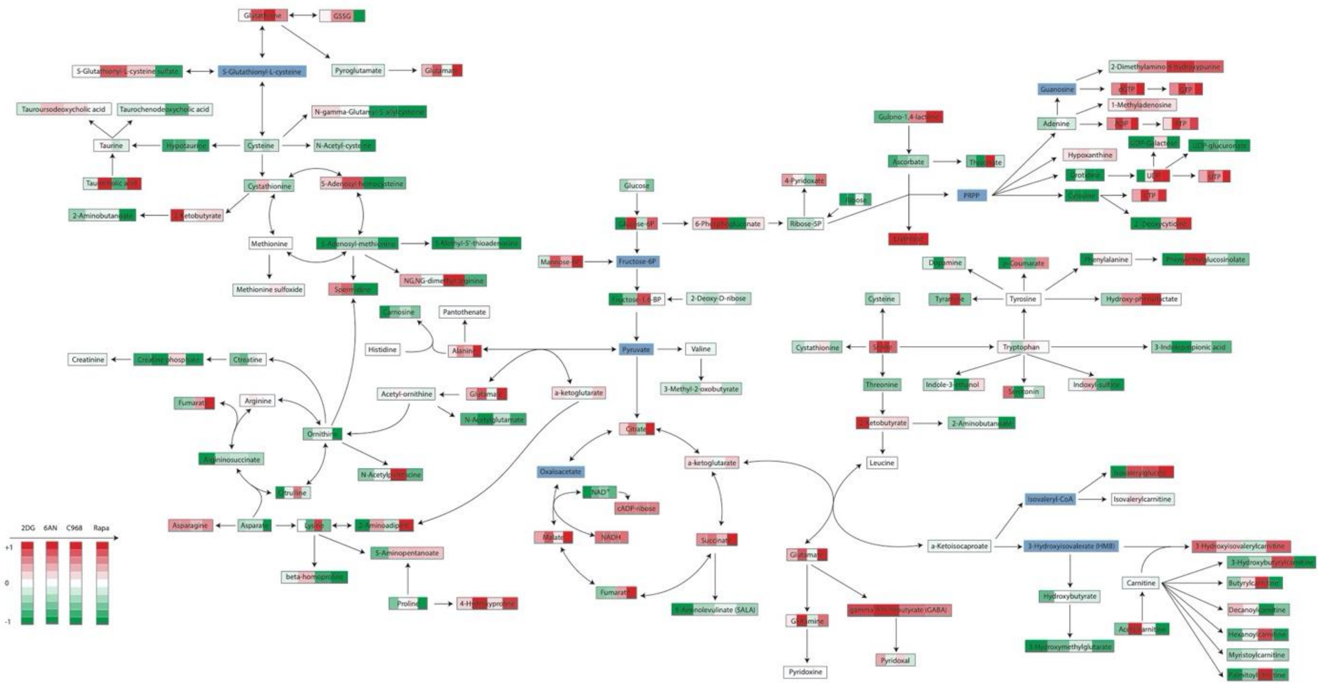

Figure S10. The metabolic flux pathway connection of 2DG, 6AN, C968 and Rapamycin treatment at 24 hours profiled by the HR-LC-MS/MS untargeted metabolomics. The metabolites are color coded based on the ^{13}C-flux ratio which calculated using formula ($flux\ ratio_{13C} = \frac{(\sum Intensity_{13C}/\sum Intensity_{12C})_{drug\ treated}}{(\sum Intensity_{13C}/\sum Intensity_{12C})_{Vehicle}}$). The increased flux is represented in *red* color, and decreased flux is represented in *green* color; and undetected metabolites are colored in *blue*.

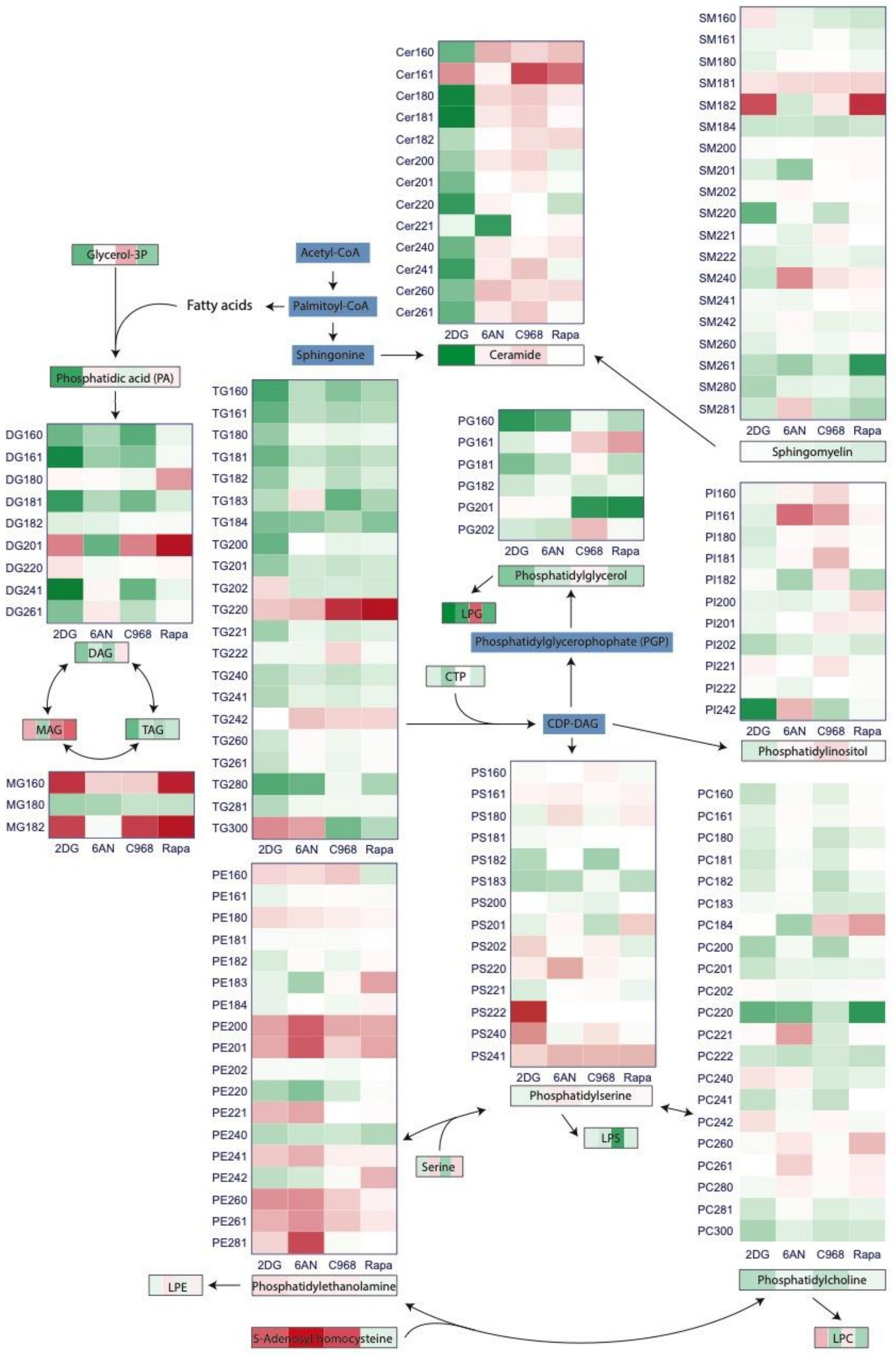

Figure S11. The lipid class flux pathway connection of 2DG, 6AN, C968 and Rapamycin treatment at 2 hours profiled by the untargeted HR-LC-MS/MS lipidomics. The lipid class are color coded based on the ^{13}C-flux ratio which calculated using formula ($flux\ ratio_{13C} = \frac{(\sum Intensity_{13C}/\sum Intensity_{12C})_{drug\ treated}}{(\sum Intensity_{13C}/\sum Intensity_{12C})_{Vehicle}}$), and deconvoluted in different fatty acid chain length lipid. The increased flux is represented in *red* color, and decreased flux is represented in *green* color; and undetected metabolites are colored in *blue*.

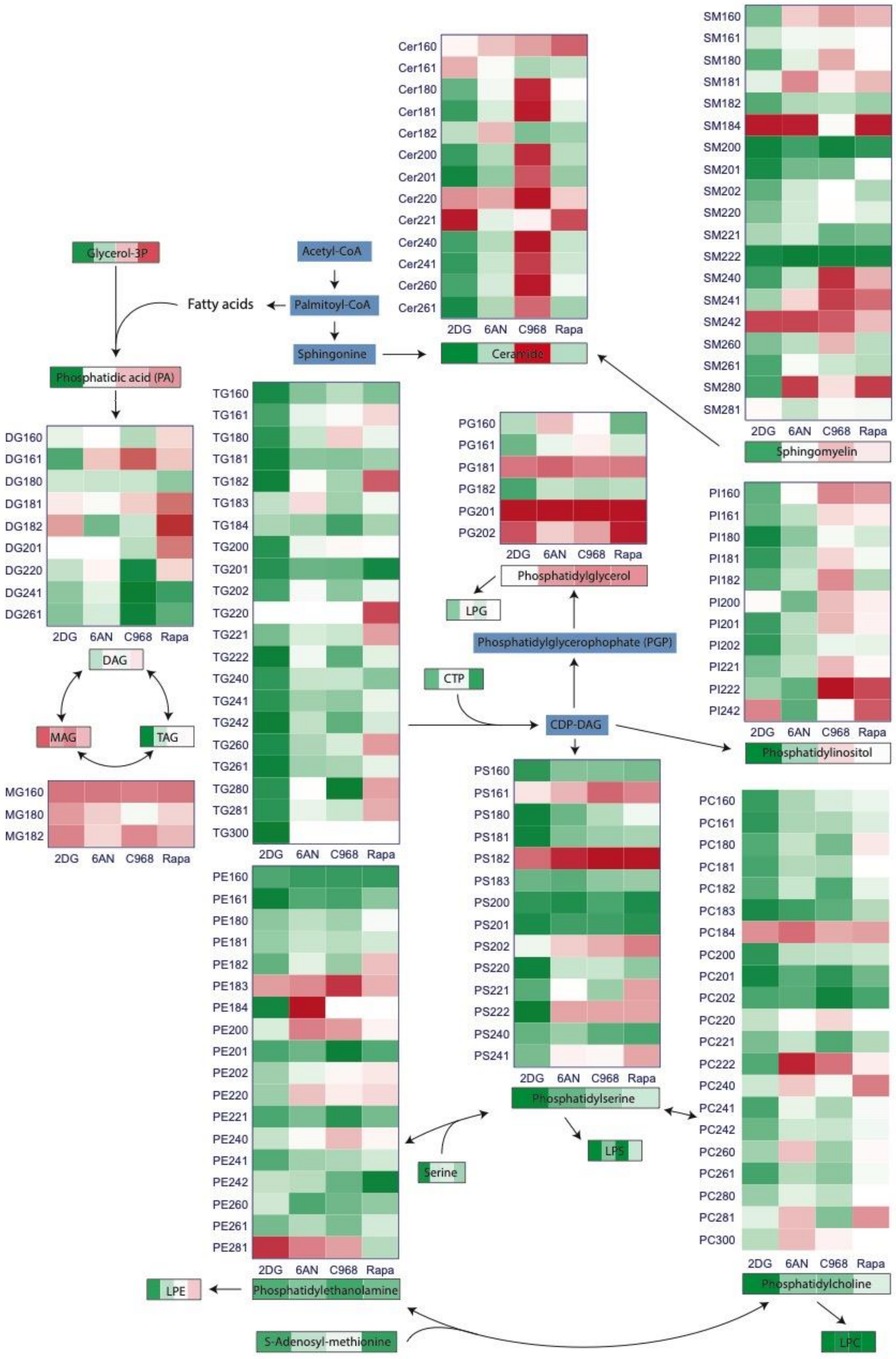

Figure S12. The lipid class flux pathway connection of 2DG, 6AN, C968 and Rapamycin treatment at 16 hours profiled by the HR-LC-MS/MS untargeted lipidomics. The lipid class are color coded based on the ^{13}C-flux ratio which calculated using formula ($flux\ ratio_{13C} = \frac{(\sum Intensity_{13C}/\sum Intensity_{12C})_{drug\ treated}}{(\sum Intensity_{13C}/\sum Intensity_{12C})_{Vehicle}}$), and deconvoluted in different fatty acid chain length lipid. The increased flux is represented in *red* color, and decreased flux is represented in *green* color; and undetected metabolites are colored in *blue*.

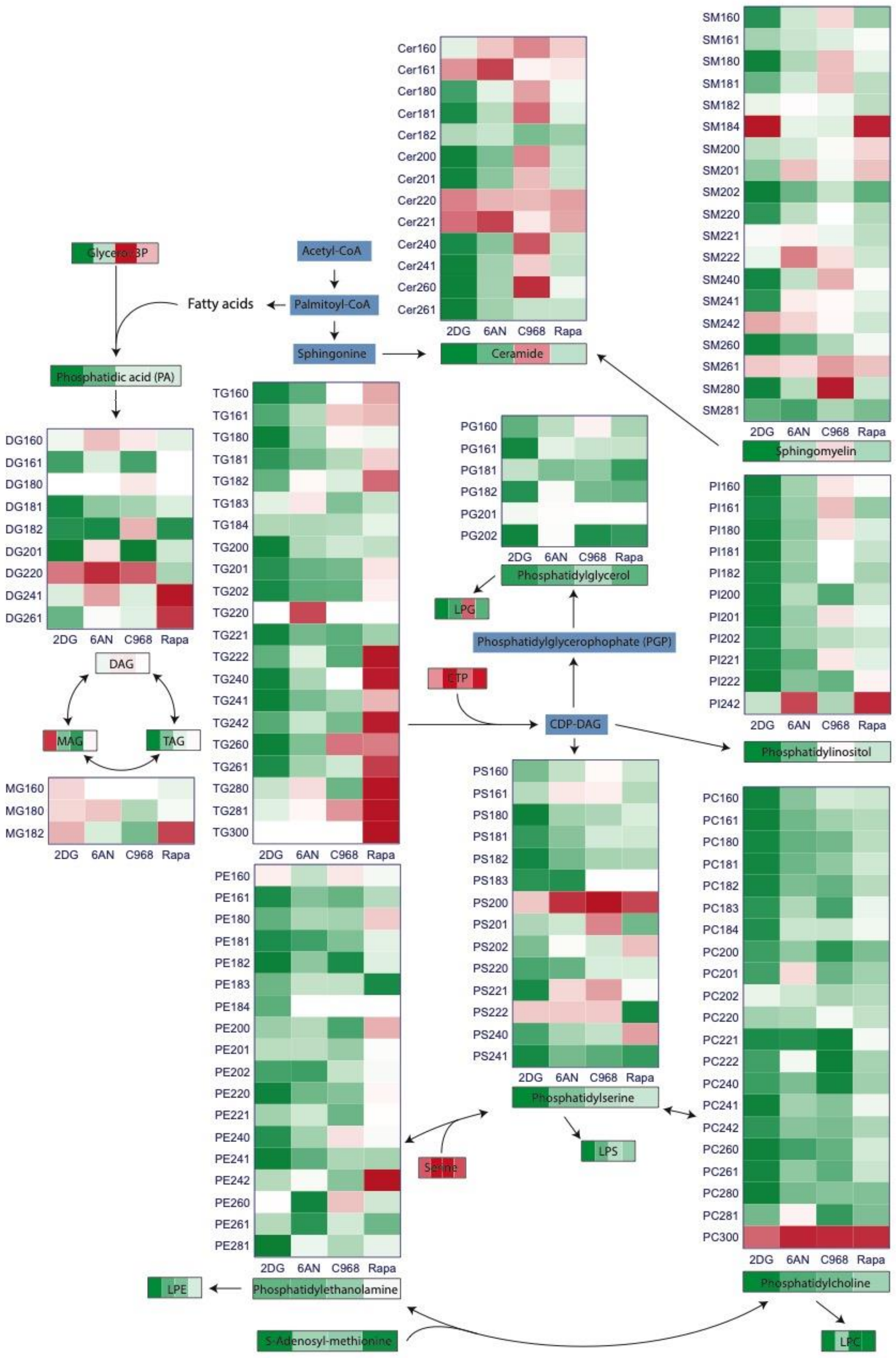

Figure S13. The lipid class flux pathway connection of 2DG, 6AN, C968 and Rapamycin treatment at 24 hours profiled by the HR-LC-MS/MS untargeted lipidomics. The lipid class are color coded based on the ^{13}C-flux ratio which calculated using formula ($flux\ ratio_{13C} = \frac{(\sum Intensity_{13C}/\sum Intensity_{12C})_{drug\ treated}}{(\sum Intensity_{13C}/\sum Intensity_{12C})_{Vehicle}}$), and deconvoluted in different fatty acid chain length lipid. The increased flux is represented in *red* color, and decreased flux is represented in *green* color; and undetected metabolites are colored in *blue*.

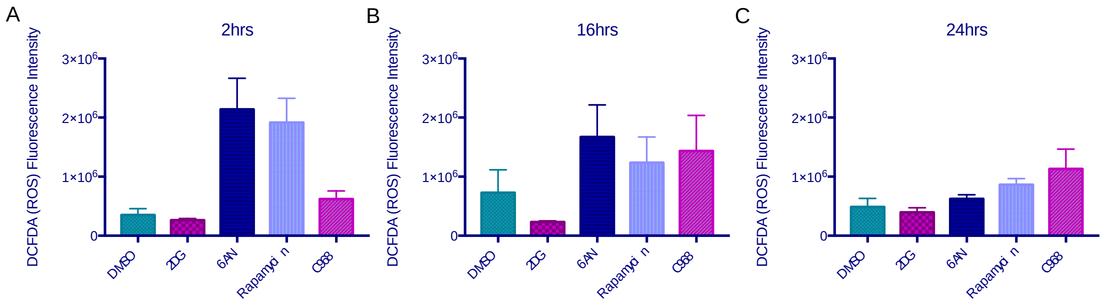

Figure S14. Reactive oxygen species (ROS) detection of MCF-7 cell treated by DMSO, 2DG, 6AN, rapamycin, and C968. After **(A)** 2-hour, **(B)** 16-hour, or **(C)** 24-hour, the fluorescent intensities of DCFDA/H2DCFDA were recorded representing the general ROS levels produced by the MCF-7 breast cancer cells.

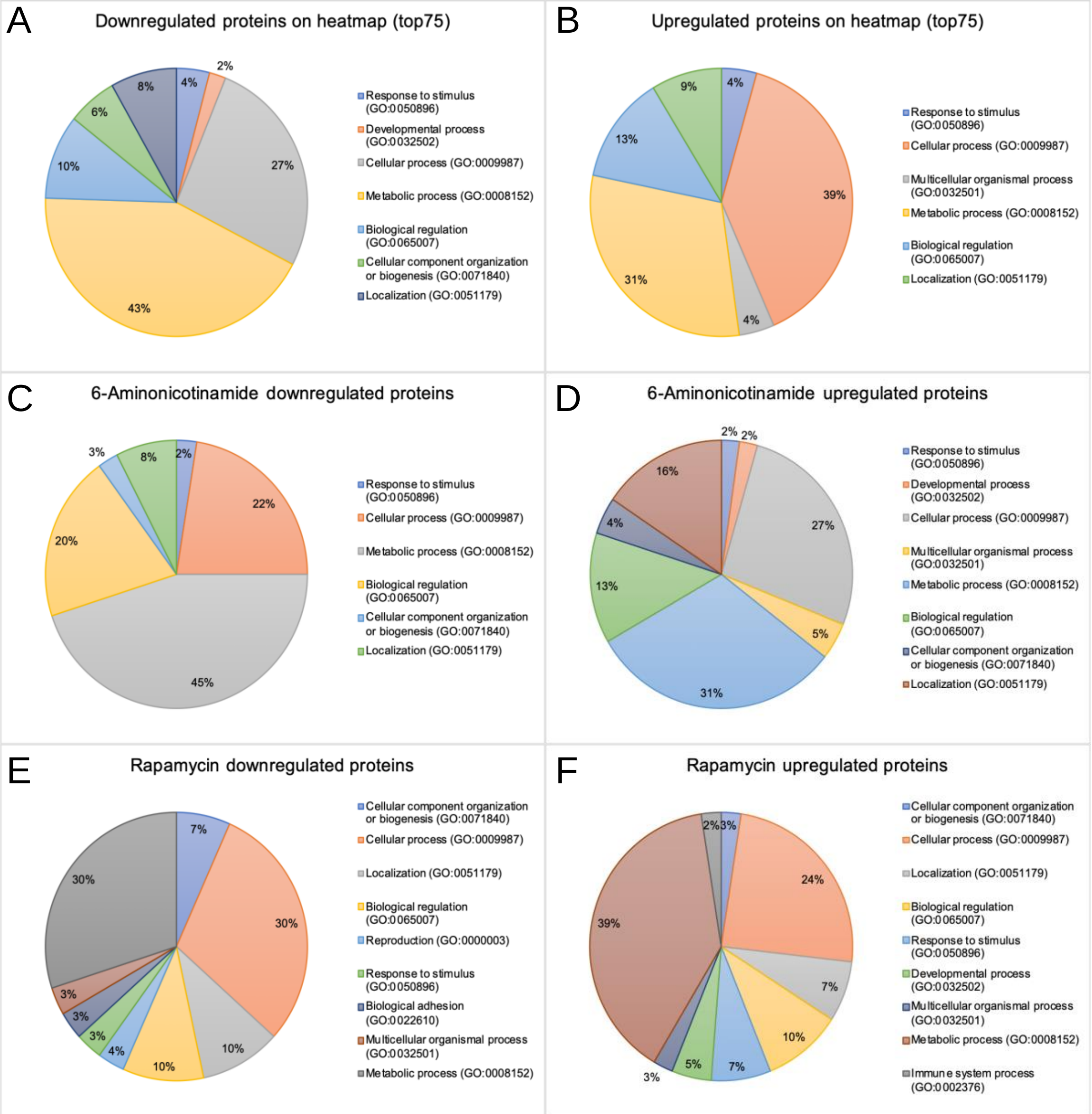

Figure S15. Enrichment gene ontology biological processes of 6AN and rapamycin treated MCF-7 cell proteins by triple SILAC and HR-LC-MS/MS. Proteins **(A)** down-regulated or **(B)** up-regulated in both 6AN and rapamycin treated top 75 dysregulated feature on heatmap are classified mainly in metabolic and cellular process. The 25 **(C)** downregulated and **(D)** up-regulated proteins in MCF-7

cells treated by 6AN at 24-hour. The 25 **(E)** down-regulated and **(F)** up-regulated proteins in MCF-7 cells treated by rapamycin at 24-hour. The proteomic GO enrichment analysis is processed by the PANTHER 14.1 https://www.pantherdb.org.

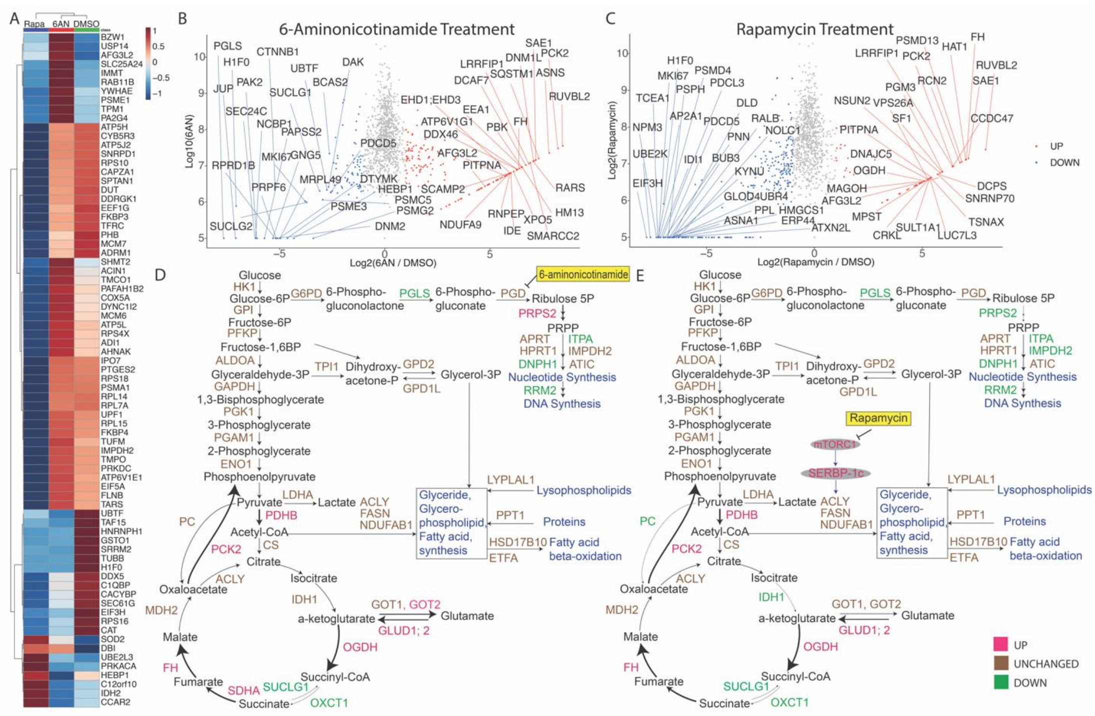

Figure S16. Protein alterations detected by stable isotope labeling by amino acids in cell culture (SILAC) associated with metabolic fluxomic results. (A) Heat map of the 75 most-changed proteins in 6AN and rapamycin vs DMSO control treated MCF-7 cells. The MA scatterplot of (B) the top 25 up-regulated (*red*) and down-regulated (*blue*) proteins induced by 24 hr 6AN treatment; and (C) the top 25 up-regulated (*red*) and down-regulated (*blue*) proteins induced by 24 hr Rapa treatment. The enzymes regulating cellular metabolism are mapped onto the metabolic flux pathways and altered by 24 hr 6AN (D) or 24 hr Rapa treatment (E). The up-regulated (fold change > 2), down-regulated (fold change < 0.5), or unchanged proteins are colored in red, green, or brown, respectively. The arrow thickness represents the relative flux ratio in the metabolic pathways.

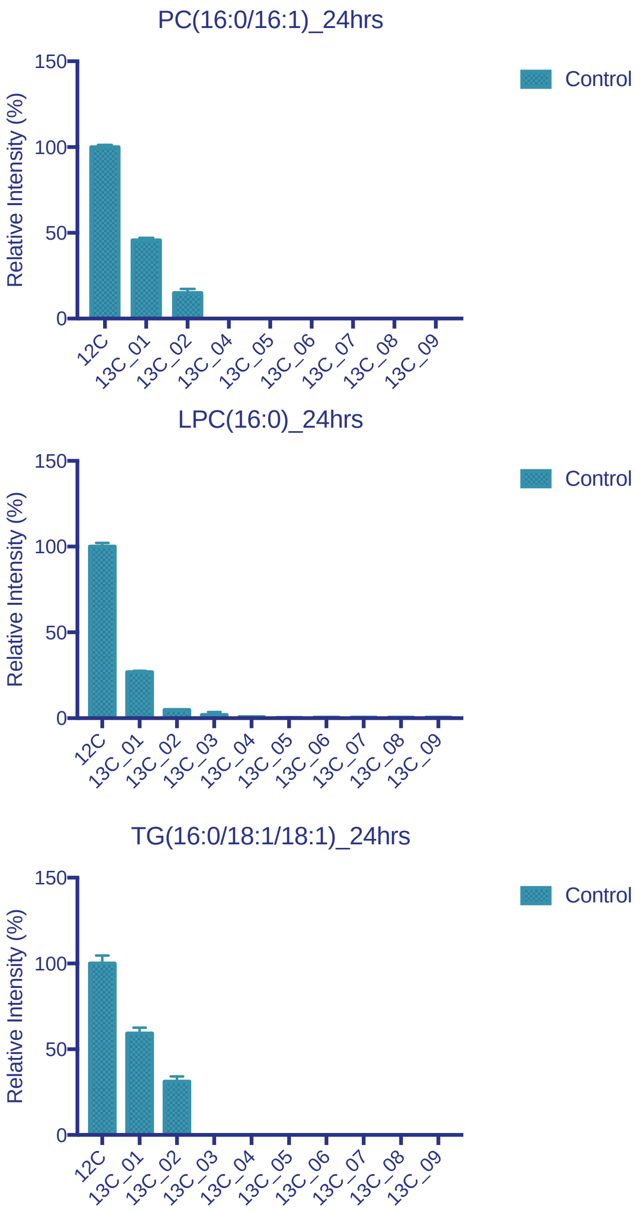

Figure S17. The ^{12}C-glucose untreated isotopologue of three lipids, PC(16:0/16:1), LPC(16:0) and TG(16:0/18:1/18:1).
